# Non-Coding RNAs in Breast Cancer: Diagnostic and Therapeutic Implications

**DOI:** 10.3390/ijms26010127

**Published:** 2024-12-26

**Authors:** Roman Beňačka, Daniela Szabóová, Zuzana Guľašová, Zdenka Hertelyová

**Affiliations:** 1 Department of Pathophysiology, Medical Faculty, Pavol Jozef Šafarik University, 04011 Košice, Slovakia; daniela.szaboova@student.upjs.sk; 2 Center of Clinical and Preclinical Research MEDIPARK, Pavol Jozef Šafarik University, 04011 Košice, Slovakia; zuzana.gulasova@upjs.sk (Z.G.); zdenka.hertelyova@upjs.sk (Z.H.)

**Keywords:** breast cancer, non-coding RNAs, long non-coding RNAs, microRNAs, piwi-interacting RNAs, circular RNAs, silencing RNAs, small nuclear RNA, small nucleolar RNA, biomarkers

## Abstract

Breast cancer (BC) is one of the most prevalent forms of cancer globally, and has recently become the leading cause of cancer-related mortality in women. BC is a heterogeneous disease comprising various histopathological and molecular subtypes with differing levels of malignancy, and each patient has an individual prognosis. Etiology and pathogenesis are complex and involve a considerable number of genetic alterations and dozens of alterations in non-coding RNA expression. Non-coding RNAs are part of an abundant family of single-stranded RNA molecules acting as key regulators in DNA replication, mRNA processing and translation, cell differentiation, growth, and overall genomic stability. In the context of breast cancer, non-coding RNAs are involved in cell cycle control and tumor cell migration and invasion, as well as treatment resistance. Alterations in non-coding RNA expression may contribute to the development and progression of breast cancer, making them promising biomarkers and targets for novel therapeutic approaches. Currently, the use of non-coding RNAs has not yet been applied to routine practice; however, their potential has been very well studied. The present review is a literature overview of current knowledge and its objective is to delineate the function of diverse classes of non-coding RNAs in breast cancer, with a particular emphasis on their potential utility as diagnostic and prognostic markers or as therapeutic targets and tools.

## 1. Introduction

Breast cancer (BC) is one of the most prevalent cancers in humans and the most prevalent cancer in women of all races worldwide (~25% incidence). According to the WHO, in 2022, estimated female breast cancer incidence was 2.3 million cases, constituting 11.6% of all cancer diagnoses, surpassing lung (9.4%) and colorectal cancer (8.9%) [1]. In 2020, 2.3 million women were diagnosed with BC and the mortality of female breast cancer has reached 685,000 deaths globally. The highest reported incidence of BC was in the northern hemisphere (Northern America, Northern and Western Europe), Australia and New Zealand (>80 per 100,000 females). In contrast, Central America, Eastern and Middle Africa, and South-Central Asia reported the lowest rates (<40 per 100,000). Melanesia, Western Africa, and Micronesia/Polynesia reported the highest mortality rates (>20 per 100,000), while most other regions in the world reported mortality rates between 10 and 15 per 100,000 [2]. More than 70% of all new cases and 81% of all deaths were observed in women aged 50 years and older. However, the main cause of BC mortality in women is not the primary tumor itself, but metastases to distant organs. Metastases are the major cause of more than 90% of breast-cancer-related deaths. The five-year survival rate for metastatic BC is only around 28%. In contrast, localized breast cancer has a very high five-year survival rate (91–99%) [3,4]. In addition to metastasis, multidrug resistance (MDR) is another common phenomenon responsible for BC-related deaths. Due to the increasing prevalence of risk factors and an aging population, the incidence of breast cancer is expected to rise from 64% to 95% in developing countries and from 32% to 56% in developed countries by 2040 [1,5].

Considerable efforts have been made to improve early diagnosis, categorization, and make treatment more efficient in the early stages before the disease progresses to a stage with a low therapeutic response. However, numerous challenges remain [3]. The primary risk factor for breast cancer is female sex and advanced age. Other potential risk factors include dense breast tissue, benign breast conditions, the earlier onset of menarche, later menopause, lack of childbearing or lack of breastfeeding, and obesity [6].

Based on the current state of knowledge, breast cancer is an inherently heterogeneous group of disorders, exhibiting distinct histomorphological and etiological features. It is characterized by the presence of genetic mutations in critical regions of the genome within somatic breast cells or cells of germinative origin. Additionally, there is growing evidence suggesting that epigenetic regulators play a pivotal role in both the initiation and progression of tumors [7,8]. Epidemiological studies show changes in the genetic and epigenetic integrity caused by environmental influences, which can be predictive or non-predictive. Epigenetic alterations, including changes to DNA and histone proteins, can either enable or disable other epigenetic regulators, such as non-coding RNAs, which, in turn, affect the expression of protein-coding genes. It is becoming increasingly evident that the role of the epigenome is extensive and multifaceted, with a vast array of effects. This has led some authors to postulate that epigenetic processes and regulators (e.g., ncRNAs) may be a key factor in the etiology of breast cancer [7,9]. In recent years, there has been a significant expansion in our understanding of epigenetic alterations in breast cancer. These data not only offer promising markers for specific BC subtypes, but also have the potential to form new therapeutic strategies. Metastasis and multidrug resistance (MDR) caused by specific mutations represent prevalent phenomena responsible for breast-cancer-related deaths in women. Given the limited number of options for targeting genes, drugs that target specific genetic and epigenetic aberrations are opening new ways of research and providing promising tools for the development of novel breast cancer treatments [9,10,11]. The objective of this review article is to present an overview of recent data from studies on the molecular dysregulation of breast cancer at the level of non-coding RNAs. A particular focus of this review is on their potential as diagnostic molecular markers and possible therapeutic tools as epigenetic regulators of oncogenes and tumor-suppressor genes [12].

## 2. Histological Types and Molecular Characteristics of Breast Cancer

**Figure 1:**
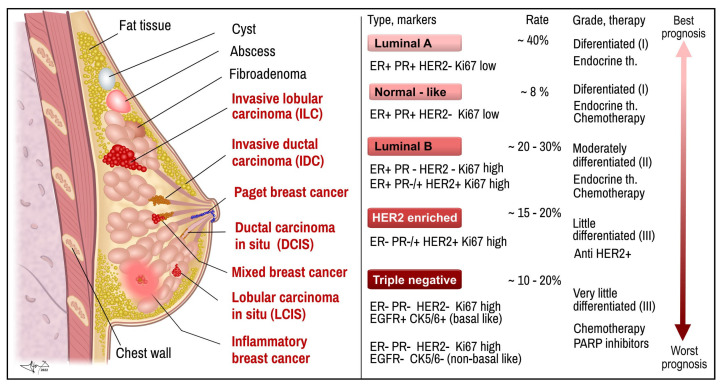
Main histological forms of breast cancer and their molecular classification. On *the left* is a schematic representation of various cell masses in the breast, including benign tumor and malignant tumor subtypes (red). Molecular subtypes of breast cancer with essential markers, histological grade, therapeutic outline, and prognosis are shown on *the right*.

Mammary tumors can arise from any cell of the mammary gland, either ductal epithelial, lobular milk-producing glandular cells, or stromal parenchymatous or even fat cells (Figure 1). Often, the tumor occurs only in one breast; approximately 2% to 5% of all tumors are bilateral breast cancers. BC can be a benign non-invasive tumor characterized by enhanced cell proliferation without anaplastic cell abnormalities, and the tumor mass grows encapsulated and strictly confined to the place of the origin. Alternatively, it can be a malignant invasive type (IDC—invasive ductal carcinoma, ILC—invasive lobular carcinoma), which is characterized by an abnormal phenotype with morphological pleiotropy, invasiveness, and metastatic spread into regional lymph nodes and/or into distant locations. Another category is carcinoma in situ, in which the tumor shows abnormal malignant-like cell morphology without signs of invasivity (DCIS or LCIS, ductal or lobular carcinoma in situ, respectively). In addition to histological subtypes of BC, other clinically defined BC subtypes are recognized, including inflammatory breast cancer (IBC). It is a fast-growing and aggressive subtype that infiltrates breast lymph vessels and the skin, and is manifested by a typical reddish inflammatory lesion and metastatic breast cancer (MBC) new colonies of cancer cells, e.g., in the lungs, bones, liver, brain, etc. [13,14].

Each tumor is morphologically and genomically different, which has an impact on different clinical manifestations of the disease, its possible resistance to a certain type of treatment, and the need for personalized therapy for each patient [12]. Based on the cellular, morphological, and molecular characteristics, breast cancer is classified into five major subtypes defined by the presence of hormone receptors (i.e., receptors for estrogen (ER), progesterone (PR)), type 2 receptors for human epidermal growth factor (HER2), and proliferation index (Ki 67). These subtypes include **luminal A, luminal B, HER2 positive enriched, normal-like, and triple-negative (basal-like)** [13,14,15] (Figure 1).

(1) **The luminal A subtype (ER+PR+ HER2(–) Ki-67(low))** is positive for hormone receptor ER and/or PR, but negative for HER2, with a low level (<14%) of Ki-67. **The normal-like subtype (ER+PR+ HER2(–) Ki-67(low))** of BC shares the same characteristics as the luminal A subtype, including a higher survival rate. However, the prognosis for the normal-like subtype is worse than that observed in the luminal A subtype [16,17,18].
(2) **The luminal B subtype (ER+PR+ HER2(+/–) Ki-67(high))** is hormone – receptor - positive (ER and/or PR) and is either HER2-positive or HER2-negative, but with high levels (≥14%) of Ki-67. The luminal B subtype has a worse prognosis and a smaller survival rate compared with the luminal A subtype [17]. This group of hormone-positive ER/PR+ cancers (HR+) constitute around 60–70% or even 75–85% of all BCs and commonly show less aggressive behavior than ER/PR (−) BC [19]. First-line treatment for the HR (+) subtype is endocrine therapy, either aromatase inhibitors or selective estrogen receptor degraders (e.g., fulvestrant) and ovarian function suppression (drugs or surgery) in postmenopausal women. Additional targeted agents (e.g., inhibitors of CDK4/6, mTOR) or immunomodulants can be used in combination with endocrine therapy. HR+ BC has the best prognosis with a median survival of about 57 months, while the median survival is about 33 months for HR(-) breast cancer [14,20,21,22].
(3) **HER2+-enriched BC (ER(−) PR(−) HER2+, Ki 67(high))** subtype is negative for hormone receptors ER and/or PR, respectively, but positive for human epidermal growth factor receptor type 2 (HER2+), and Ki67 index rates are high. The HER2+ subtype accounts for ~20% (15–25%) of all the BC subtypes [23] and approx. the same for ovarian cancers (25%) and 18% of gastric cancers [24,25]. Before the era of modern therapy, this subtype provided the worst prognosis and lowest survival rate of BC [26,27]. Trastuzumab (the first HER2-targeted humanized monoclonal antibody) used alone or in combination with other chemotherapeutics has substantially improved the therapeutic response, time to relapse, and overall prognosis of HER2+ [27], although the therapeutic response was often variable, and was accompanied by primary and secondary resistance to the treatment (~30–40%) [28]. This leads to the wider use of newer HER2-targeting drugs such as monoclonal antibodies such as pertuzumab or trastuzumab, new bispecific monoclonal antibodies ZW25 (Zanidatamab) or KN026, which bind two distinct domains of HER2 [29], antibody–drug conjugates such as T-Dxd (trastuzumab deruxtecan), trastuzumab emtansine (T-DM1) [25], inhibitors of immunosuppression (monoclonal IgG4 antibody against PD-1’ Pembrolizumab), or inhibitors of cyclin-dependent kinases [30,31].
(4) **Triple-negative BC (TNBC**) **or basal-like BC (ER−, PR−, HER2 (−), Ki-67(high))** is recognized by the absence of ER, PR, and HER2− (ER−/PR− and HER2−) [32]. TNBC accounts for 10–20% of all BC cases and is the most heterogeneous and the most aggressive subtype with a high rate of recurrences and metastases with poor five-year survival rates [14]. The TNBC subtype occurs in younger women and has a higher prevalence in women of African or African American ethnicity [14,17,32,33]. TNBC can be further subdivided into basal-like subtypes and non/basal tumor-specific subtypes. Lehmann et al. recognize even more molecular subtypes: basal-like 1 (BL1) and 2 (BL2), mesenchymal (M), and one luminal androgen receptor group (LAR) [34]. The phenotypes of TNBC, which is frequent among young patients, include large size and advanced tumor grade. TNBCs often metastasize to local and distant lymph nodes at diagnosis and exhibit high proliferative rates [28,32].

## 3. Non-Coding RNAs in Breast Cancer

It is well established that over 75% of the human genome is transcribed into RNA, yet only 2% to 5% encodes proteins. DNA not responsible for the synthesis of proteins is transcribed into a range of non-coding RNAs (ncRNAs), which engage in numerous cellular activities and are crucial for cell growth, maintenance, and differentiation. In essence, ncRNAs can be classified according to their length into long ncRNAs (lncRNAs) with a length of >200 nucleotides and into small ncRNAs with a length of ≤200 nucleotides. Moreover, small ncRNAs can be classified into several subclasses, including microRNAs (miRNAs), piwi-interacting RNAs (piRNAs), small interfering RNAs (siRNAs), small nuclear RNAs (snRNAs), small nucleolar RNAs (snoRNAs), small cytoplasmic RNAs (scRNAs), transfer RNAs (tRNAs), and ribosomal RNAs (rRNAs) [35,36,37].

ncRNAs have been shown to play a crucial role in transcription, post-transcriptional processing, and translation [38]. ncRNAs are dysregulated in a variety of human disorders, including cancers and neurological and immunological disorders. A substantial body of evidence has elucidated the involvement of ncRNAs in a multitude of cellular processes, including proliferation, migration, invasion, apoptosis, and the stemness of cancer cells, as exemplified by breast cancer. Some ncRNAs may undergo overexpression or functional upregulation in cancer, potentially contributing to tumor promotion and their classification as oncogenes. Conversely, the levels of some ncRNAs may be reduced or absent, and their effects may be diminished or insufficient, which could classify them as tumor suppressors. Nevertheless, the traditional categorization of these molecules does not always align with their actual biological effects in specific cancers. For example, some ncRNAs may be overexpressed while their effect in cancer is inhibitory; other ncRNAs may utilize different pathways in different cancers. The current view is that there is an interconnected network of ncRNAs in which multiple ncRNAs may function as competitive endogenous RNAs (ceRNAs) [39]. For example, lncRNAs can act as miRNA inhibitors, competitively binding and inhibiting the expression of miRNAs with tumor-suppressive effects [40]. It should be noted that any particular ncRNA represents only a minor component in a giant orchestrated action, which includes myriads of genetic alterations and epigenetic dysregulations [41].

### 3.1. Long Non-Coding RNAs

**Figure 2:**
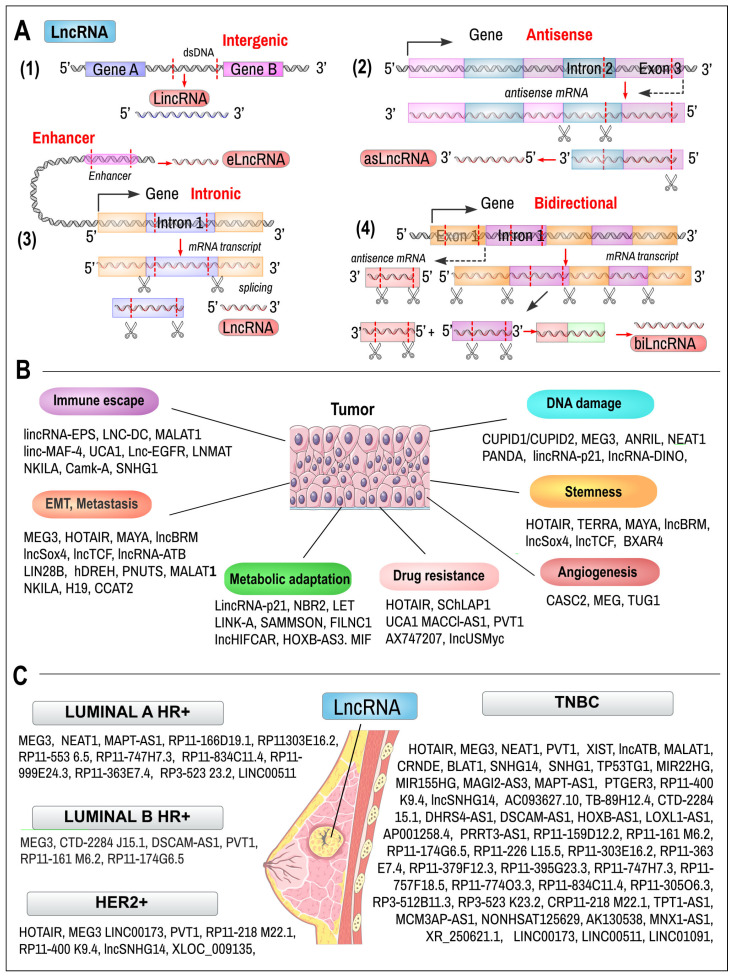
Biogenesis of long non-coding RNAs (LncRNAs) and their role in breast cancer. (**A**) (1) Intergenic RNAs (LincRNAs) are transcripts of dsDNA between two protein-coding genes. (2) Antisense LncRNAs (asLncRNAs) are transcribed from complementary strands, either within the intronic or exonic region of protein-coding genes. (3) Intronic LncRNAs are transcripts of dsDNA from the intronic region of a protein-coding gene. Enhancer LncRNAs (eLnc RNAs) are transcribed from the dsDNA of enhancer regions of genes. (4) Bidirectional LncRNAs (biLncRNAs) originate from the bidirectional transcription of protein-coding genes. LncRNAs mediate the positioning of transcription factors in the promoters of protein-coding genes. (**B**) LncRNAs associated with different cellular processes that are important in cancerogenesis. (**C**) LncRNAs associated with each molecular subtype of breast cancer. Data are based on several sources [47,50,51,52,61,62].

***Long non-coding RNAs (lncRNAs)*** are a class of single-stranded non-coding RNAs, typically longer than 200 nt, operating in the nucleus and cytoplasm and interacting with RNA, DNA, and proteins. lncRNAs can act post-transcriptionally, serving as a sponge for different miRNAs [37,38,39,42,43]. LncRNAs can be transcribed from intergenic dsDNA, from exonic and intronic DNA, or composed from both. They were shown to be produced from transcriptional pseudogenes or mitochondrial genes [44]. Several tumor suppressor genes generate long antisense ncRNAs [45]. Current data from the GENCODE database indicate that the human genome contains ~16,000 lncRNA genes that encode more than 28,000 distinct lncRNAs [46,47] (Figure 2A).

***Function***. LncRNAs play a role in the epigenetic regulation of gene expression, either at the transcriptional or post-transcriptional level [48,49]. To be more precise, lncRNAs can perform the following functions: (a) act as molecular switches, orchestrating specific transcriptional activities at a particular location, time, or developmental stage, and/or in response to specific stimuli; (b) act as fine-tuning regulators of transcriptional activity, controlling transcription factors, enhancers, and miRNAs; (c) act as guides to navigate proteins into ribonucleoprotein complexes or serve as scaffolds that assemble multiple proteins interacting with RNAs [45]. Among the various types of ncRNAs, lncRNAs demonstrate a remarkably high degree of specificity in tissue expression, rendering them potential diagnostic targets. The recent advancement of molecular technologies enabled the precise identification of the functions and the connections of lcnRNAs with other ncRNAs within regulatory RNA network [38,39,40].

***LncRNAs in breast cancer.*** A substantial body of evidence indicates that lncRNAs play a pivotal role in the multistage process of breast cancer tumorigenesis, including growth, invasiveness, metabolic adaptation, DNA damage repair, angiogenesis, the development of malignant epithelial-mesenchymal transition (EMT), and the formation of metastatic phenotypes. LncRNAs exert a significant influence on the efficacy of the immune system control over cancer survival, and their alterations allow for an immune escape as well as multidrug resistance. Further, mutated or dysregulated lncRNAs contribute to cancer cell stemness and immortality (Figure 2B) [38,43,49,50].

Altered levels of lncRNAs were observed in tumor tissues as well as in surrounding tissues and can be detected in blood samples from patients with a different molecular subtype of BC as well as other cancers [47]. lncRNA *TERRA* (Telomeric Repeat-containing RNAs) has been demonstrated to exert a negative regulatory effect on telomerase [51]. Aggressive forms of BC such as HER2+ and TNBC tumors are characterized by the overexpression of lncRNA *HOTAIR* (HOX antisense intergenic RNA), which is a spliced and polyadenylated trans-acting RNA derived from the antisense transcript of the HoxC gene (locus 12q13.13). It maintains a high degree of sequence identity (95–99%) in all primates. *HOTAIR* interacts with key epigenetic regulators, including histone methyltransferase PRC2 (Polycomb Repressor Complex 2) and histone demethylase LSD1, inducing the epigenetic silencing of numerous tumor suppressor genes (e.g., TP53) and ncRNAs (e.g., miR-7) [52]. It plays an important role in the regulation of the cell cycle. *HOTAIR* is misregulated in several types of cancers and contributes to proliferation, epithelial-mesenchymal transition (EMT), tumor migration, and invasion [53]. In human BC, this lncRNA is the primary oncogenic promoter and prometastatic lncRNA [54].

The effects on EMT and metastasis are conveyed via different pathways, including those of transforming growth factor β, Wnt/β-catenin, and PI3K/AKT/MAPK/mTor [55]. The knockout of *HOTAIR* has been demonstrated to reduce drug resistance in breast cancer cells to doxorubicin [56]. Maternally expressed gene 3 (*MEG3*) is an imprinted long non-coding RNA (lncRNA) gene (locus 14q32.2) that is expressed maternally and functions as a tumor suppressor. It is located within the *DLK1-MEG3* imprinting region of human chromosome 14. *MEG3* functions as a growth inhibitor in tumor cells by activating p53 machinery [57]. It is downregulated (inactivated or mutated) in most human cancers, including BC (e.g., squamous cell carcinoma in the head, neck, esophagus, hepatocellular carcinoma, neuroblastoma, lymphomas, meningiomas, osteosarcoma, ovarian and cervical cancer, melanoma, etc.) [57,58]. Its expression is associated with tumor size and infiltrating and metastatic capabilities.

There is a potential correlation between the level of *MEG3* and the expression of miRNAs miR-182 and miR-29 in BC [59]. In contrast, lncRNAs such as *ANCR*, *MALAT1*, and *NKILA* have been demonstrated to inhibit tumor growth and attenuate the invasive spreading and metastasis of BC [60]. A correlation has been identified between PAM50 subtyping and the expression of lncRNAs in breast cancer tissues. The expression of these lncRNAs is highly specific, with the majority acting as “enhancers” [61,62].

As anticipated, different roles of lncRNAs can be found in subtypes of BC (Figure 2C). Several studies identified differentially expressed lncRNAs in HR+ and HR− BC subtypes. In the HR+ subtypes, lncRNAs such as *RP11-53O19.2*, *RP11-473 L15.3* [61], *LINC01297* [62], *lncRNA-DLEU1* [58], *LINC01016*, *SIAH2-AS1* [63], *RUSC1-AS1*, *SNHG3* [64], *FAM83H-AS1* [65], *MIAT* [66,67], and *AP000439.3* [57] were overexpressed, whereas *HOXA-AS2*, *MEG3* [68], *RP11-303E16.2* [62], *MORT* [69], and *GSN-AS1* [65] were reported as downregulated. As recently demonstrated, lncRNAs such as *HOTAIR*, *BCAR4*, and *linc-ROR*s may play a role in the metastatic phenotype of BC [39,60]. The mechanisms underlying the invasive and metastatic clones within the tumor suggest the inhibition of several tumor suppressor or metastasis suppressor genes, indicating that lncRNAs may act as “metastatic promoters”. The HR-negative/HER2+ BC subtype and TNBC (basal-like) represent 15–20% and 15% of invasive BCs, respectively, and both are associated with worse prognosis and are considered more aggressive than HR+ subtypes [70]. Upregulated *lncRNA LOC339535* (referred to as LINK-A) activating normoxic HIF1 alpha signaling allows cell clones to withstand hypoxia in TNBC and is inversely associated with the survival of these patients [71]. A number of lncRNAs have been identified as being significantly upregulated in HR-negative/HER2+subtype of BC. These include *lncSNHG14* [72], *lncATB* [73], *AK130538* [55], *LINC00511* or *MNX1-AS1* [74]. In contrast, expression of certain lncRNAs has been observed to be downregulated. These include *NONHSAT125629*, *XR_250621.1*, *XLOC_l2_001548* [55] or *MEG3*, *MIR22HG*, *RP11- 305O6.3*, *LINC01091*, *XLOC_009135*, and *MAGI2-AS3* [74].

LncRNAs are recognized as having possibly the highest impact of all ncRNAs on the resistance to chemotherapeutics. The high expression of lncRNAs such as *lncSNHG14* and *lncATB* in HER2+ patients has been correlated with resistance to trastuzumab. *LncSNHG14* activates the NRF2 signaling pathway by inducing the expression of PABPC1 by the modulation of H3K27 acetylation in the promoter region of the PABPC1 gene [72]. *LncATB* affects trastuzumab resistance via TGF-β signaling, by binding to miR200c, upregulating ZEB1 and ZNF-2017, and inducing EMT [74].

Further reviews of a comprehensive scope examine the prospective utility of lnc-RNA as a diagnostic indicator and its potential applications in the treatment of BC [75].

### 3.2. MicroRNAs

**Figure 3:**
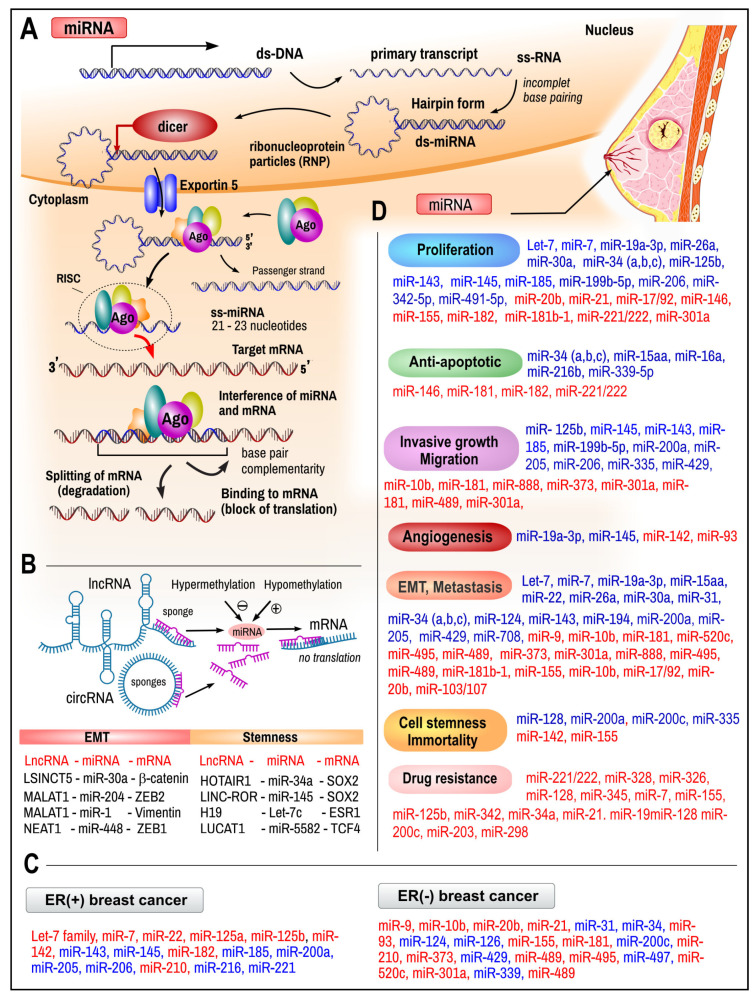
Biosynthesis and use of microRNA in breast cancer. (**A**) The primary transcript of microRNA (pri-miRNA) is processed in the nucleus into pre-miRNA by the RNAse III and self-assembles into double-stranded RNA with a small loop (hairpin shape). miRNA is exported from the nucleus into the cytosol (exportin 5), where a hairpin loop is cut off by Dicer endoribonuclease (RNA helicase), while the rest of the miRNA assembles into a complex called RNA-induced silencing complex (RISC) together with the Argonaute family of nucleoproteins (Ago). RISC binds to complementary motifs in the mRNA to cause post-transcriptional mRNA silencing by blocking the translation of the mRNA or degrading the mRNA into fragments. (**B**) Examples of upregulated (red) or downregulated (blue) miRNAs in ER+ and ER- breast cancer. (**C**) Regulatory role of miRNA in gene expression can be achieved by i) binding to mRNA and preventing translation or ii) binding to sponges made by lncRNA and/or circRNA. The synthesis of miRNAs can be epigenetically regulated by hyper- or hypomethylation. (**D**) Upregulated (red) and downregulated (blue) miRNAs in breast cancer, further subdivided according to their function in different cellular processes. Schematic visualization of acquired data on different miRNAs is based on the sources mentioned in the text and Table 1.

***MicroRNAs (miRNAs)*** are a class of evolutionarily conserved (more than 1000 miRNA may exist) small (typically 21–23 nucleotides in length) single-stranded multifunctional ncRNAs which are transcribed from miRNA coding dsDNA segments located in exons of protein-coding genes (about 70%) or from non-coding DNA within intergenic regions [76]. The biogenesis of microRNAs starts in the nucleus. miRNA genes are transcribed by polymerase II into long primary transcripts (pri-miRNA), which are later arranged into typical c hairpin structure, which is recognized and cleaved by the RNAase III family enzyme Drosha into precursor miRNA (pre-miRNA) that contains 70–100 nucleotides with interspersed mismatches and a hairpin structure (Figure 3A). The complementation of bases (C–G; A–U) in miRNA is not always perfect, leaving several mismatched bases. Pre-miRNAis is actively exported (Ran-GTP, Exportin 5) into the cytoplasm where it is cleaved by the RNAse III Dicer into double-stranded miRNA molecules. In the following step, strands are separated by helicase into the mature single-stranded microRNA and antisense-microRNA. The final single-stranded microRNA can then be incorporated into three possible complexes, miRISC (miRNA-containing RNA-induced silencing complex), miRNP (miRNA-containing ribonucleoprotein complex), or miRgonaute, and functions as a gene regulator at the post-transcriptional level [77,78]. RISC binds to complementary motifs in the mRNA to cause post-transcriptional mRNA silencing by (1) prolonging binding and blocking the translation of the mRNA or (2) degrading the mRNA strand by splicing it into fragments.

***Function.*** MiRNAs play an integral role in the regulation of biological processes through their post-transcriptional regulation of gene expression and repression of the translation of targeted mRNAs. They accomplish this through three main mechanisms: cleavage, marking for degradation, and pairing with targeted mRNA molecules through perfect base complementarity at the 3′ untranslated regions [76,79].

The loss of miRNA function can be attributed to a multitude of mechanisms, including genetic mutations, deletions, epigenetic silencing, and alterations in miRNA processing. The activity of miRNA molecules can be modified by methylation, resulting in either activation or deactivation (Figure 3B). miRNAs may be overexpressed, underexpressed, or dysfunctional, resulting in oncogenic (prometastatic) or tumor-suppressive (anti-metastatic) outcomes, respectively [80]. LncRNAs and circulatory RNAs (circRNA) can act as miRNA sponges, reducing their regulatory effect over mRNAs. This provides an extra layer of complexity in the miRNA–target interaction network. The specific interactions within the ncRNA network between long non-coding RNAs (lncRNAs), microRNAs (miRNAs), and messenger RNAs (mRNAs) enable breast cancer cells to undergo epithelial-mesenchymal transition (EMT) or to establish a cancer stem cell (CSC) phenotype, as illustrated in Figure 3B. Examples of interactions between circRNAs and miRNAs and target mRNAs are given later.

***miRNAs in breast cancers***. MicroRNAs have been extensively studied in the context of various cancers based on their involvement in tissue development [81], cell proliferation, differentiation [80], and apoptosis [82]. The connection between dysregulated microRNAs and breast cancer was first observed by Iorio et al. in 2005 [83]. During their study of 76 breast cancer and 10 normal breast samples, they confirmed the significant deregulation of 29 miRNAs and a set of 15 miRNAs capable of correctly distinguishing the tumor from normal breast tissue with 100% accuracy. The most consistently upregulated microRNAs were miR-21 and miR-155, and the downregulated ones were miR-10b, miR-125b, and miR-145 [83]. Blenkiron et al. have identified 133 microRNAs expressed in normal and tumor breast tissue out of 309 unique human microRNAs [84]. Different genes are affected by different subgroups of miRNAs; each epigenetic action has an impact on tumor development, tumor characteristics, and behavior. Downregulated tumor suppressor miRNAs are unable to act against oncogenes, and upregulated oncogenic miRNAs act by inducing genomic instability, causing DNA repair system dysfunctionalities and, therefore, promoting proliferation, angiogenesis, migration, invasion, and metastasis and inhibiting apoptosis [85]. An overview of the various microRNAs and their roles in the pathogenesis of BC is provided in Table 1.

*miR-10b* is highly expressed in metastatic breast cancer cells. It has been proven that miR-10b promotes migration, metastasis, and invasion via directly targeting and suppressing the synthesis of HOXD10 protein and Syndecan-1. HOXD10 is a negative regulator; its suppression results in an expression of pro-metastatic genes RhoC, alfa3-integrin, MT1-MMP, and urokinase plasminogen activator receptor (uPAR). miR-10b expression is induced by the transcriptional factor Twist; therefore, higher levels of Twist are also connected with metastatic potential [86,87].

*miR-9* is significantly upregulated in distant metastatic loci rather than in primary breast cancer tumors. It has been shown that miR-9 enhances the ability of the tumor to metastase and gives tumor cells stem-cell-like properties, advancing them in their growth, motility, and invasiveness. miR-9 acts via directly targeting CDH1 (E-cadherin), FOXO1, STARD13, and LIFR. The suppression of CDH1, FOXO1, and STARD13 results in epithelial-mesenchymal transition (EMT) and increased motility and invasiveness. The inhibited expression of CDH1 also leads to angiogenesis via the activation of the beta–catenin signaling pathway and the elevated expression of vascular endothelial growth factor (VEGF). miR-9 expression is induced by the activity of MYC, MYCN, and PDGRβ [88,89,90].

*miR-21* is significantly upregulated in advanced-stage metastatic breast cancer tumors. The high expression of miR-21 promotes EMT, proliferation, invasion, metastasis, and radioresistance by suppressing the expression of PTEN and TPM1. PTEN promotes proliferation, invasion, and radioresistance via AKT activation. The suppression of PDCD4 results in ELF4e activation, c-jun activation, an increase in CDK1 expression, and a decrease in p21 expression, leading to reduced apoptosis and promoted cell proliferation; the suppression of MARKs leads to increased cell motility and the suppression of TIMP3; and Maspin enhances invasion and metastasis. miR-21 expression is induced by the activity of STAT3, NFkB, and ERK1/2 [91,92,93].

*miR-155* upregulation in breast cancer tumor cells has been correlated with recurrent tumors even after chemotherapy and radiotherapy. MiR-155 inhibits the expression of RhoA, SOCS1, FOXO3a, and TRF1, resulting in increased cell survival, EMT and proliferation, chemoresistance, and radioresistance. Suppressed RhoA leads to EMT promotion, TRF1 to genomic instability, and SOCS1 to the activation of JAK1/STAT signaling and, therefore, increased proliferation and metastasis. The suppression of FOXO3a results in increased levels of antiapoptotic and decreased levels of proapoptotic proteins, and apoptosis inhibition occurs [93,94].

*miR-181* can act as either onco miRNA or tumor suppressor miRNA; its character depends on targeted genes. Acting as an onco miRNA, miR-188 directly targets and suppresses the expression of *BIM* (apoptosis inhibition, resistance to anoikis), *ATM* (DNA repair system dysfunction), and *PDCD4* (increased proliferation). The suppression of PDCD4 results in ELF4e and c-jun activation and the expression of CDK1 and p21. Acting as a tumor suppressor, miR-188 suppresses the expression of BCL-2 (resulting in apoptosis) and MMP-14, and *PHLDA1* (resulting in metastasis inhibition) [95,96,97].

*Let-7* is downregulated in breast cancer tumor cells. In normal cells, let-7 acts as a tumor suppressor, ensuring the inhibition of proliferation and metastasis via its target genes. Let-7 suppresses the expression of CDK6, IGF2BP1, and cyclin D1 to inhibit proliferation. The suppression of HMGA1 leads to decreased levels of cyclin B1 (proliferation inhibition) and TGF-beta signaling inhibition (metastasis and EMT inhibition). The suppression of CCR7 leads to metastasis and invasion inhibition. In cancer cells, the downregulation of let-7 causes abnormal cell proliferation, growth, and the acquirement of invasive character [98,99].

*miR-34* has tumor suppressing abilities; in normal cells, miR-34 inhibits proliferation and induces apoptosis, cell cycle arrest, and senescence. miR-34 acts by directly targeting numerous genes involved in cell cycle regulation. The suppression of LMTK3, MDM4, CDK4/6, cyclin D1, CDC23, PRKCA, Src, and LDHA results in proliferation inhibition. It has a negative effect on the viability of cancer cells and can stop metastasis formation. The suppression of FRA1, SNAIL1, TWIST1, and ZEB1 has an effect on EMT inhibition. miR-34 inhibits stemness potential via the suppression of NOTCH 1/4, apoptosis via BCL2, and proliferation and migration via AXL. miR-34 forms a positive feedback loop with tumor suppressor p53. TP53 gene is activated by DNA damage (radiation, oxidative stress, chemotherapy); abundant p53 during normal conditions is destabilized and degraded. Damage leads to the stabilization and accumulation of p53 in the cells, resulting in cell cycle arrest and apoptosis. miR-34 is the direct target for p53; therefore, increased levels of p53 lead to increased levels of miR-34. miR-34 targets SIRT1 and suppresses its expression. Suppressed SIRT1 expression further promotes p53 expression and activity, resulting in the inhibition of proliferation and induced apoptosis [100,101,102].

*miR-125* acts as a tumor suppressor microRNA, reduces cell proliferation, and inhibits metastasis. miR-125 directly targets ARID3B and HER2+, and suppressing their expression results in metastasis inhibition. The suppression of HuR, KIAA1522, and MKNK2 leads to the inhibition of proliferation and metastasis formation [103,104].

*miR-145* inhibits cell proliferation and metastasis formation and induces apoptosis by directly targeting and suppressing ER-α expression. EMT inhibition is a result of Oct4 suppression and, therefore, the inhibition of SNAIL, ZEB1, and ZEB2 expression. The suppression of c-myc leads to p21 expression and proliferation and metastasis inhibition. miR-145 also directly targets and suppresses Fascin, JAMA, MUC1 (metastasis inhibition), and VEGF and NRAS (angiogenesis inhibition) [105,106,107].

**Table 1:** Micro RNA in breast cancer. (Arranged according to identification number). Abb. Exp. expression level in breast cancer.

Micro-RNA	Cytogen. Locus	Exp	Function of microRNA in Breast Cancer Cells	Important mRNA Targets	Citation
**Let-7** **family**	Different location	↓	Tumor suppressor role: Anti-metastatic, Anti-proliferative	RAS, HMGA2, CDK6 Cyclin D1, CCR7	[108,109,110,111,112]
**miR-7**	9q21.32	↓	Tumor suppressor role:Pro-apoptotic, Anti-metastatic, Anti-proliferative	FAK, REG γ HOXB3, PAK1	[113,114,115,116]
**miR-9**	1q22	↑	Oncogenic role: Metastasis, Angiogenesis	CDH1 (E-cadherin)	[88]
**miR-10b**	2q31.1	↑	Oncogenic role: Invasion, Migration, Metastasis	HOXD10 Syndecan-1	[117,118]
**miR-20b**	Xq26.2	↑	Oncogenic role: Proliferation, Metastasis	PTEN	[119]
**miR-21**	17q23.2	↑	Oncogenic role: Proliferation, Invasion, Radioresistance, Metastasis	PTEN, TPM1 PDCD4, TIMP3, BCL2	[120,121,122,123,124]
**miR-22**	17p13.3	↓	Tumor suppressor role: Anti-metastatic	CDK6 SIRT1 SP1	[125,126,127]
**miR-31**	9p21.3	↓	Tumor suppressor role: Anti-metastatic, Anti-proliferative, Anti-invasive, Pro-apoptotic	PKC ε, Smad3/4 GNA13, SATB2	[128,129,130,131]
**miR-34**	1p36.22	↓	Tumor suppressor role: Pro-apoptotic, Anti-proliferative Anti-metastatic, Anti-stemness	BCL-2, SIRT1, MDM4 CDK4/6, Cyclin D1 AXL NOTCH1/4	[132,133,134,135,136,137]
**miR-93**	7q22.1	↑	Oncogenic role: Metastasis, Angiogenesis	LATS2	[138]
**miR-124**	8p23.1	↓	Tumor suppressor role: Anti-metastatic	Slug CDK4	[139,140]
**miR-125a**	19q13.41	↓	Tumor suppressor role: Anti-proliferative	HDAC4 HuR	[140,141]
**miR-125b**	11q24.1	↓	Tumor suppressor role: Anti-metastatic	HER2	[142,143]
**miR-126**	9q34.3	↓	Tumor suppressor role: Anti-proliferative, Anti-metastatic, Anti-angiogenic	IGFBP2 PITPNC1 MERTK VEGF	[144,145,146]
**miR-142**	17q22	↑	Oncogenic role: Growth, Metastasis	APC	[147]
**miR-143**	5q32	↓	Tumor suppressor role: Anti-proliferative, Anti-metastatic	HER3	[148]
**miR-145**	5q32	↓	Tumor suppressor role: Anti-metastatic, Anti-proliferative, Anti-angiogenic	ER-α, c-Myc, Oct4 MUC1, JAMA VEGF, N-RAS	[105,106,149,150,151,152]
**miR-155**	21p21.3	↑	Oncogenic role: Proliferation, Metastasis, Genome instability	RhoA, SOCS1 FOXO3a TRF1	[153,154,155,156]
**miR-181**	1q32.1	↑	Oncogenic role: Proliferation, DNA repair dysf., Anti-apoptotic	PDCD4 ATM, BIM	[157,158]
**miR-182**	7q32.2	↓	Tumor suppressor role: Pro-apoptotic	BRCA1	[159]
**miR-185**	22q11.21	↓	Tumor suppressor role: Anti-proliferative	E2F6, DNMT1	[160]
**miR-200a**	1p36.33	↓	Tumor suppressor role: Anti-metastatic, Self-renewing	SLUG ZEB2	[161,162]
**miR-200c**	12p13.31	↓	Tumor suppressor role: Anti-metastatic, Self-renewing	BMI1 ZEB1	[163,164]
**miR-205**	1q32.2	↓	Tumor suppressor role: Pro-apoptotic, Anti-metastatic, Anti-proliferative	HER3 E2F P53	[165,166,167]
**miR-206**	6p12.2	↓	Tumor suppressor role: Anti-metastatic, Anti-proliferative	ERα CyclinD2	[168,169]
**miR-210**	11p15.5	↑	Oncogenic role: DNA repair dysf.	RAD52	[170]
**miR-216**	2p16.1	↓	Tumor suppressor role: Pro-apoptotic	P2X7	[171]
**miR-221**	Xp11.3	↓	Tumor suppressor role: Epithelial-mesenchymal transition	ADIPOR1	[172]
**miR-301a**	17q22	↑	Oncogenic role: Metastasis	PTEN	[173]
**miR-335**	7q32.2	↓	Tumor suppressor role: Anti-metastatic, Pro-apoptotic	SOX4 Bcl-w	[174,175]
**miR-339**	7p22.3	↓	Tumor suppressor role: Pro-apoptotic	Bcl-6	[176]
**miR-373**	19q13.42	↑	Oncogenic role: Metastasis	CD44	[177]
**miR-429**	1p36.33	↓	Tumor suppressor role: Anti-proliferative, Anti-metastatic	CRKL	[178]
**miR-489**	7q21.3	↑	Oncogenic role: Metastasis	E-Cadherin, Smad3	[179]
**miR-495**	14q32.31	↑	Oncogenic role: Metastasis	E-Cadherin, REDD1	[180]
**miR-497**	17p13.1	↓	Tumor suppressor role:Pro-apoptotic, Epithelial-mesenchymal transition, Growth inhibition, Anti-proliferative	Slug BCL-W, RAF1 Cyclin E, Cyclin D	[181,182,183,184,185]
**miR-520c**	19q13.42	↑	Oncogenic role: Metastasis	CD44	[177]

There are currently a number of other systematic review articles that collect and analyze available knowledge about the expression, functions, and diagnostic or therapeutic potential of microRNAs [186,187].

### 3.3. Circular RNAs

***Circular RNAs (circRNAs)*** are a class of single-stranded ncRNAs (19–25 nt) with typical covalently closed circular structures without 5′ and 3′ ends and poly-A tail. Due to this enclosed continuous structure, circRNAs possess high stability against exonuclease-mediated degradation. circRNAs were originally discovered in the 1970s and the current number of known transcripts is more than 400,000. Most circRNAs (84%) are transcribed from protein-coding genes via RNA: Poly II (i.e., exones (ecircRNAs), introns (i-icircRNA), or both (ei-circ RNA), or fusion circRNA (f-circRNAs) by ‘back-splicing of pre-mRNAs’ as alternative splicing connecting the 3′splice end of the downstream exon to the 5′splice site of the upstream exon [188]. In addition to the nuclear circRNAs, mitochondrial circRNAs appear to exist as well [189,190,191,192].

**Figure 4:**
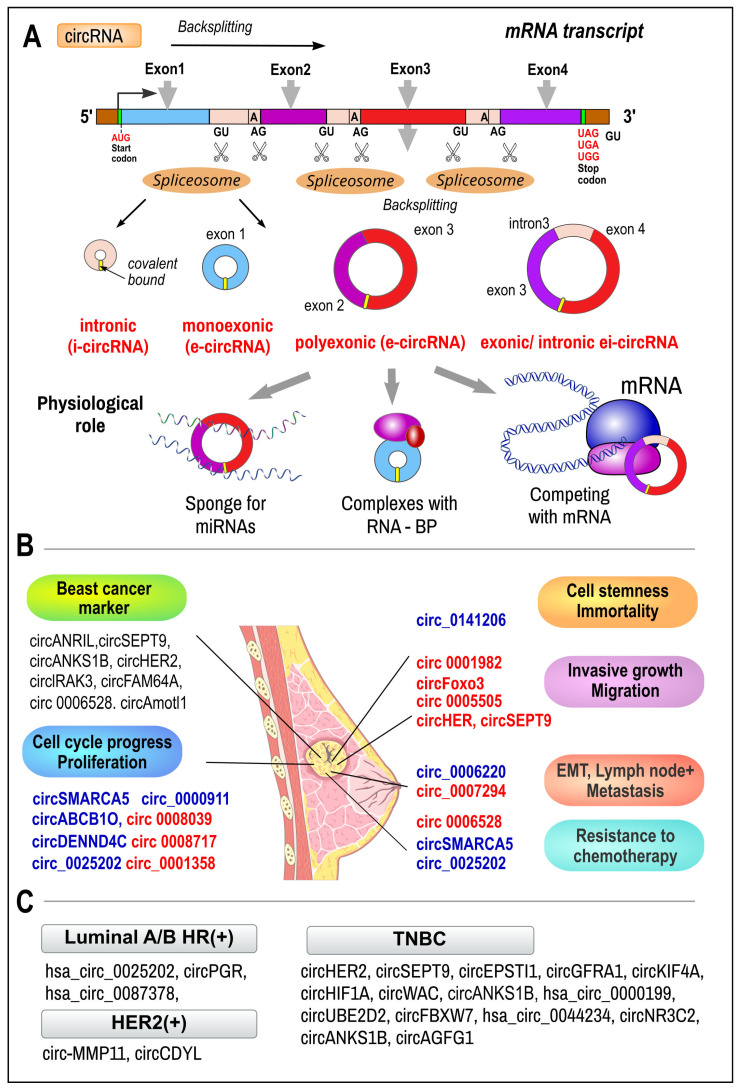
Biogenesis and dysregulation of circRNAs in breast cancer. (**A**) CircRNAs can originate from intronic (i-circRNA), exonic (e-circRNA), or both intronic and exonic (ei-circRNA) transcripts of protein-coding genes. These transcripts can undergo either direct back-splitting (e.g., in e-circRNA formation) or the debranching of resistant intron variants (e.g., i-circRNA formation), intron-pairing-driven circularization, or exon skipping (e.g., ei-circRNA formation). CircRNA can act as an miRNA sponge by binding and suspending RNA-BPs (RNA binding proteins), or by interfering with mRNA (messenger RNA) translation. (**B**) CircRNAs associated with pro-oncogenic activity (*red*) and tumor suppressor activity (*blue*) and their role in breast cancer pathogenesis. The same circRNA can fall into several categories. (**C**) Distribution of circRNAs dysregulated in different BC subtypes (luminal A/BHR (+), hormone-positive, HER2+, and TNBC types). Image of breast tumor adapted from Servier Medical Art under license CC-BY-3.0.

***Function.*** CircRNAs can regulate gene transcription, likely through several ways: (a) By acting as ’sponges’ to suspend specific miRNAs and prevent them from binding to their target mRNAs. circRNA thus serves as an endogenous miRNA regulator (inhibitor). (b) circRNAs can also bind to nucleoproteins, which can participate in miRNA and siRNA processing or chromatin remodeling. (c) circRNA can compete with mRNA and block translation by binding to rRNA nucleoproteins. (d) circRNAs are important contents in exosomes, which can regulate peripheral cell functions, thus influencing the tumor microenvironment [189,192] (Figure 4A).

***circRNAs in breast cancer.*** Principally, circRNAs can be involved in cancer pathogenesis in two ways: (a) acting through miRNA sponge, circRNAs may serve as competitive inhibitors of miRNA and behave as oncogenes, and (b) acting through the miRNA sponge pathway or by other mechanisms, circRNAs may behave like tumor suppressors. circRNAs are highly evolutionarily conserved across species and the knowledge of circRNA obtained in experimental animals can be transferred to humans. Figure 4B illustrates the involvement of circRNAs in the diverse characteristics of breast cancer cells.

One of the first described roles of circRNA in BC was circDENND4C, which is an HIF1α-associated circRNA promoting the proliferation of breast cancer under hypoxia [193]. Furthermore, circRNAs exhibit good tissue- and stage-specific expressions, and also show long-term stability against degradation in tissue and the blood. These characteristics make circRNAs promising biomarkers in tumor diagnostics. Competitive endogenous circRNAs (denoted as ce-circRNAs) serve in breast cancer progression [190,191,192,194]. In progressive tumors, exosomal *circHIPK3* released from tubular BC cancer cells modulates angiogenesis within the tumor by sponging and inhibiting the action of miR-124-3p on the MTDH gene in endothelial cells on the tumor periphery [194]. Different miRNA is used in pathway circHIPK3/miR-488/MTDH in prostatic cancer progression and metastasis [195].

Similar to other non-coding RNAs, different circRNA expression patterns can be observed in ER+ versus ER- breast cancer subtypes (Figure 4C) [196]. A study by Wang et al. showed that estrogen-induced *circPGR* is upregulated in ER+ cell lines, while it serves as a sponge for tumor suppressoric miR-301a-5p [194]. Such a measure leads to the overexpression of *CDK1*, *CDK6*, and *CHEK2* (cell cycle regulators), and, ultimately, promotes the growth, progression, migration, and colony formation of ER+ breast cancer cells [197]. Hsa_circ_0025202 [198] and hsa_circ_0087378 [198] are other ER+ subtypes that have been reported to be downregulated in tumors. Hsa_circ_0025202 possibly acts as an miR-182-5p sponge, affecting FOXO3a activity and potentially suppressing tumor growth and enhancing tamoxifen efficacy if upregulated. The downregulation of hsa_circ_0025202 has been negatively correlated with lymphatic metastasis and the histological grade of the primary lesion [198]. Additionally, studies have identified the differential expression of circRNAs in both the ER-negative/HER2+ and TNBC subtypes. circ-MMP11 [199] and circCDYL [200] were upregulated in HER2+ tumors and act as sponges for miR-153-3P and miR-92b-3p, respectively. circ-MMP11 promotes breast cancer progression and increases resistance to lapatinib via the inhibition of miR-153-3p and regulation of ANLN expression in TNBC [199]. circCDYL is highly expressed in HER2+ and HER2- breast cancer tissues. In HER2- breast cancer, circCDYL acts as miR-1275-ULK1 sponge affecting ATG7 expression and promoting cancer cell proliferation [201]. In HER2+ tissues, miR-92b-3p inhibits the cell proliferation of HER2+ BC cells via circCDYL degradation [200,201,202].

Recently, circRNAs were identified as useful indicators of the progression and metastatic potential of TNBC [203]. In TNBC and the surrounding tissue, several circRNAs were found to be upregulated such as circ-HER2 [202], circ-SEPT9 [204], circEPSTI1 [205], circ-GFRA1 [206], circ-KIF4A [207], circ-HIF1A [208], circ-WAC [209], hsa_circ_0000199 [210], circUBE2D2 [211], and circANKS1B [212]. On the contrary, other circRNAs were shown to be downregulated, i.e., circFBXW7 [213], hsa_circ_0044234 [214], and circNR3C2 [215]. The dysregulation of normal circRNA patterns promotes tumor growth and distant metastasis by affecting cell proliferation, viability, migration, apoptosis, and drug resistance. Again, in most of these effects in TNBC, circRNAs serve as regulatory ceRNA through the inhibition of miRNAs’ action on target mRNA transcripts. The upregulation of circUBE2D2 in TNBC patients has been associated with advanced TNM stage, lymph node metastasis, and poor prognosis because circUBE2D2 promotes cell proliferation, actives the circUBE2D2/miR-512-3p/CDCA3 axis, and increases doxorubicin resistance in cancer cells [211]. The low expression of circFBXW7 has been associated with poor clinical outcomes as circFBXW7 expression is negatively correlated with tumor size and lymph node metastasis. The overexpression of circFBXW7 inhibits cell proliferation via the circFBXW7/miR-197-3p/FBXW7 axis [213]. The circNR3C2/miR-513a-3p/HRD1/Vimentin axis proposed by Fan et al. [215] negatively regulated the metastasis of TNBC via enhancing the tumor-suppressing properties of HRD1 [203]. The upregulation of circFBXW7 and circNR3C2 may be a possible therapeutical approach for aggressive forms of BC. CircGFRA1 and GFRA1 act as ceRNAs in TNBC by regulating miR-34a [206]. E2F1/EIF4A3-induced circSEPT9 could regulate the expression of Leukemia Inhibitory Factor (LIF) via sponging miR-637 and activating the LIF/Stat3 signaling pathway involved in the progression of TNBC [204]. Circ_0000199 facilitates the chemo tolerance of TNBC by interfering with miR-206/613-led PI3K/Akt/mTOR [185]. CircNR3C2 promotes the HRD1-mediated tumor-suppressive effect in TNBC via sponging miR-513a-3p [215]. circFBXW7 inhibits malignant progression by sponging miR-197-3p and encoding a protein of 185 amino acids in TNBC [213]. CircKIF4A regulates the progression of TNBC through miR-375 [207]. Abtin et al. recently successfully tested an exogenous siRNA-based therapy to inhibit the highly overexpressed circ_0009910/miR-145-5p/MUC1 axis in invasive HER2-negative BC metastasizing into lymph nodes [216]. Examples of regulatory pathways, e.g., *circRNA–miRNA–mRNA*, that are supposed to play critical role in BC are listed in Table 2.

In light of the pivotal role of circRNAs in tumorigenesis, numerous other review articles have been published with the aim of collating and analyzing knowledge about circRNAs, thereby contributing to a more comprehensive understanding of this complex issue. The most recent examples include systemic reviews by Huang et al. and Wang et al. [217,218].

### 3.4. Piwi-Interacting ncRNAs

**Figure 5:**
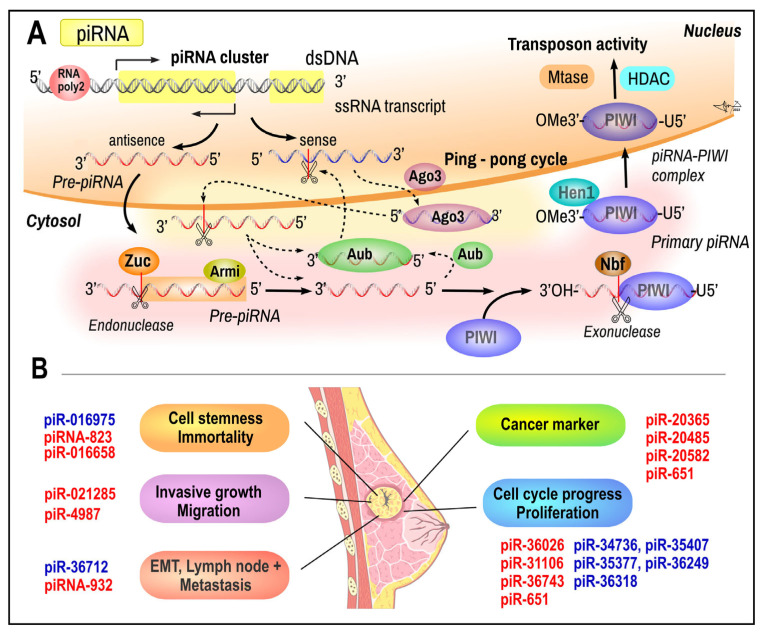
Biosynthesis of piRNAs and their alterations in breast cancer. (**A**) Formation of piRNA (PIWI-interacting RNA) occurs within and outside of the nucleus. Following transcription from genomic loci that contain transposon fragments, cluster transcripts are spliced into piRNA precursors (pre-piRNAs). The DNA loci responsible for producing piRNA precursors yield either single- or double-stranded molecules (sense and antisense transcripts). In the subsequent phase of piRNA biosynthesis (only the antisense precursor is illustrated in the figure), pre-piRNAs are transported to the perinuclear space (nuage) in close proximity to the mitochondria, where they are processed by the RNA helicase Armitage (Armi). Following despiralisation, the 5′ end of the precursor molecule is cleaved by the endonuclease Zucchini (Zuc), with the resulting 5′ fragment incorporated into PIWI proteins. The 3′ to 5′ exonuclease Nibbler (Nbr) then trims the piRNA to its final length. Concurrently, the small RNA 2′-O-methyltransferase Hen1 methylates the 2′-hydroxy group at the 3′ end. This process represents the primary biogenesis of piRNA (in the figure, this process is shown in red). The secondary biogenesis of piRNAs is called the ping-pong cycle and allows for amplification (arrows with dashed line; the DNA sequence with highlighted yellow background). The protein Aubergine (Aub) binds to antisense piRNAs and the complex cleaves sense piRNA precursors to give rise to sense piRNAs, which then form a complex with Ago3 (Argonaute3). The Ago3/piRNA complexes, in turn, cleave antisense piRNA precursors into pieces that form a complex with Aub. This cycle produces a large number of piRNAs in a short period of time. The piRNA-PIWI complexes return to the nucleus. The piRNA-PIWI complexes carry out their transposon-active activity with the help of DMTs (DNA methyl transferases) and HDACs (histone deacetylases). (**B**) Upregulated (*red*) and downregulated (*blue*) piRNAs in BC. Breast cancer image was adapted from Servier Medical Art under CC-BY-3.0 license. Data adapted from [222,228,230].

***Piwi-interacting RNAs (piRNAs)*** are a class of endogenous single-stranded ncRNAs with 26–32 nt length that associate with piwi family proteins and are specifically expressed and enriched in mammalian germ cells [219,220]. piRNAs are transcribed by RNA polymerase II from clusters of relatively short ncDNA genomic loci for piRNAs on chromosomes 17, 5, 4, and 2. Primary transcripts are exported into the perinuclear space to be processed into smaller pieces (maturation of piRNAs) by endo- and exonucleases in a Dicer-independent manner and are generated either by primary synthesis and/or through so-called ping-pong amplification [219,220,221,222] (Figure 5A). The piRNAs received their name due to the interaction with PIWI proteins (P-element-induced wimpy testis proteins), a germline-specific Argonaute family of nucleoproteins that exist at least in two isoforms, *PIWI1* and *PIWI2*. The overexpression of PIWI1 is associated with cell cycle arrest and the overexpression of PIWI2 is associated with anti-apoptotic signaling and cell proliferation [220,223,224]. Currently, piRNAs constitute one of the largest known classes of ncRNAs.

***Function.*** piRNAs may be involved in embryonic development, the maintenance of germline DNA integrity, the silencing of translational repression, the formation of heterochromatin, and the epigenetic regulation of sex determination. piRNAs can be transmitted into maternal cells and may be involved in maternally derived epigenetic effects [225]. The exact mechanism of piRNA action still remains elusive in detail. The PIWI protein with the polycomb group proteins (PcGs) interacts together into a complex that binds to genomic PcG response elements in order to act as a regulatory factor. piRNAs/PIWI complexes silence target genes at the post-transcriptional and epigenetic levels, respectively. This is similar to what happens in retrotransposons transcription (most piRNAs are antisense to transposon sequences). Transposons may lead to DNA double-strand breaks, chromosomal duplication, deletion, translocation, and inversion [224].

Insertions in the exon hinder the coding sequence: (a) the insertion into introns may change splicing patterns, and insertion in the promoter or enhancer spoils transcription; (b) at the transcriptional level, piRNAs and piwi proteins directly modify chromatin structure and histone proteins in the nucleus via the regulation of DNA methyltransferase (DNMT); (c) piRNAs are involved in cancer regulation by altering the expression of cancer-related genes in a mechanism similar to miRNA and were shown to be regulated by transcriptional factors in human cancer [226,227,228].

***piRNA in breast cancer***. Recent data provided evidence that the upregulation or downregulation of different piRNAs could be associated with various non-cancerous diseases as well as cancers of different kinds and tissue origins and their specific alteration [9,225,229,230]. Hundreds of piRNAs have been identified in different histological forms of human breast cancer involved in several processes including growth and proliferation, cell mobility and invasiveness, progression, and metastatic potential (Figure 5B). A recent study also identified 415 piRNA sequences from the medium of the luminal subtype of human breast cancer cell MCF-7; 27 piRNAs showed deregulation by pro-oncogene cyclin D1 [231]. The current list of piRNAs that are upregulated or overexpressed in breast cancer cells includes piR-651, piR-1282, piR-21131, piR-23672, piR-31106, piR-32745, and piR-36743. Conversely, the expression of piR-23662, piR-26526, piR-26527, piR-26528, piR-30293, piR-34377, piR-34736, piR-35407, piR-36318, and piR-36249 is significantly reduced in breast cancer cells [232]. Only a few of the identified piRNAs including piR-4987, piR-021285, piR-823, piR-932, piR-36712, piR-016658, and piR-016975 have been determined to possess regulatory functions, and, thus, may serve as potential biomarkers for the diagnosis or monitoring of BC treatment efficacy [222,229]. Concerning these specific piRNAs, several observations can be made. For example, Fu et al. [233] demonstrated that the upregulation of piR-021285 can modulate the invasiveness of human BC through the methylation of the gene for pro-invasive Rho GTPase - activating protein 11A (ARHGAP11A). Furthermore, the upregulation of piRNA-823 and piR-016658 and the downregulation of piR-016975 have been observed to promote cancer cell stemness. The oncogenic overexpression of piR-823 has been observed in luminal breast cancer cells. piR-823 has been demonstrated to upregulate the expression of methyltransferases DNMT1, DNMT3A, and DNMT3B. Subsequently, DNMT3B facilitates DNA methylation and the suppression of the APC (adenomatous polyposis coli) tumor suppressor, thereby activating the Wnt signaling pathway and cancer cell stemness [234].

The upregulation of piR-932 and downregulation of piR-36712 have been observed to promote EMT and metastatic processes due to the silencing of several genes through DNA methylation. PiR-932 is significantly overexpressed in the majority of progressive BC cells [235]. It may be presumed that, under normal conditions, piR-36712 may suppress the proliferation of BC cells by inhibiting selenoprotein W1 (SEPW1), which, in turn, inhibits the expression of p53 and p21 by inducing mRNA degradation. The downregulation of *piR-36712* has been observed to result in a shorter survival period, an increased risk of axillary lymph node metastasis, and a reduction in the efficacy of chemotherapeutic agents in BC [236].

The presence of lymph node metastases in progressive forms of BC is strongly associated with the overexpression of piR-20365, piR-20485, and piR-4987 [237]. The expression of piR-651 and piR-823 is increased in both breast and prostate cancer cell lines. While *piR-651* is involved in estrogen regulation, piR-823 exerts influence over the WNT/catenin pathway [234]. Additionally, the piRNAs, including piR-4987, piR-20365, piR-20485, and piR-20582, were found to be upregulated in samples from breast cancer patients when compared to matched non-tumor tissues [237]. The downregulation of piR-FTH1 has been demonstrated to target FTH1 mRNA, thereby promoting the chemosensitivity of cancer cells [238].

Piwi-like (Piwil) genes, including Piwil1, Piwil2, Piwil3, and Piwil4, have been identified in a range of cancerous tissues, including renal cell carcinoma and BC. Piwil1 overexpression is reported in BC most frequently. Piwil2 works with piR-932 to regulate cell proliferation and invasion in BC. The overexpression of Piwil2 frequently occurs alongside an increase in piRNAs, which function as an oncogene. Piwil4 expression was found to be elevated in both native BC tissues and in the in vitro MDA-MB-231 breast cancer cell line. The knockdown of Piwil4 in MDA-MB-231 BC cells led to a significant reduction in cellular migration and proliferation [235,239]. The current state of knowledge regarding piRNAs is rapidly evolving [240].

### 3.5. Small Interfering RNAs

**Figure 6:**
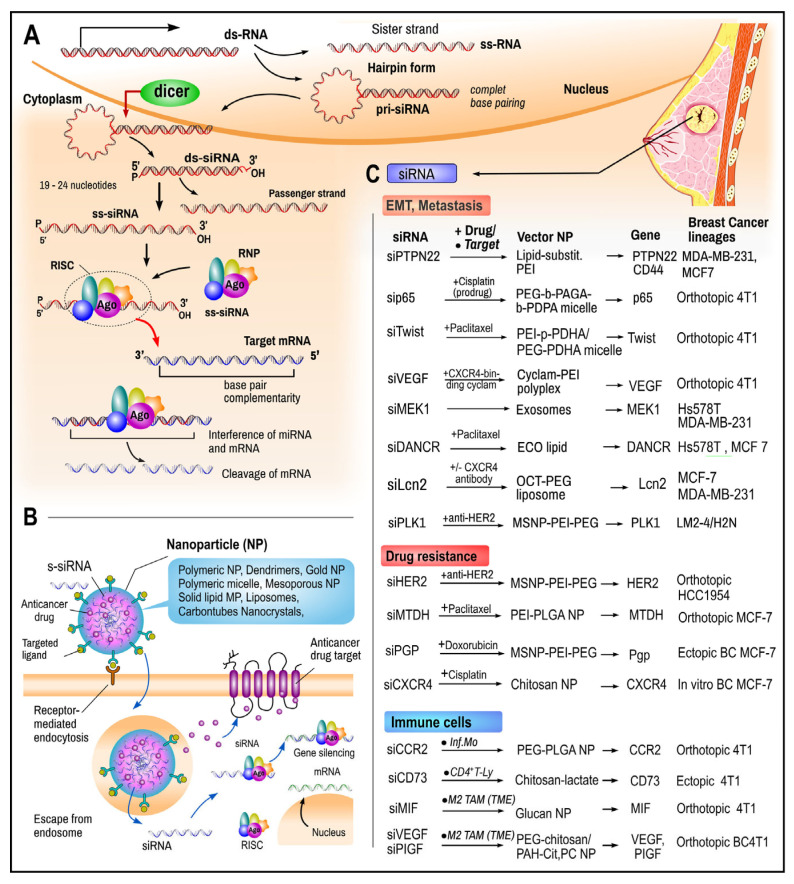
Synthesis and use of siRNAs in breast cancer. (**A**) The precursors of siRNAs are long pieces of ds RNA or hairpin ss-RNA called pri-siRNAs. In cytosol, the molecule is cleaved by the Dicer enzyme into double-stranded siRNA (ds-siRNA) and, later, single-stranded siRNA (ss-siRNA), which form a RISC complex (RNA-induced silencing complex) together with RNPs (ribonucleoproteins) and Ago protein (Argonaute family). Inside the complex, siRNA binds to complementary motifs of the target mRNA to process the fragmentation. (**B**) Nanoparticles (NPs) (types in blue box) protect siRNAs from degradation, improve targeting and increasing the accumulation of s-siRNA in the tumor cells. Receptor-mediated endocytosis is initiated by the targeting of ligands or cationic components of NP. Following an endosomal escape, s-siRNA binds to the RISC, which enables the identification and degradation of complementary mRNA targets. (**C**) Experimental use of s-siRNA to target and inhibit mRNAs of the selected genes. For an explanation, see the text. Data adapted from Ngamcherdtrakul and Yantasee [247] and other resources [246,247,248,249,250,251,252,253,254,255,256,257,258,259,260,261,262]. *Abb.* CCR2, C Motif Chemokine Receptor 2; CXCR4, CXC Chemokine Receptor 4; DOPC, 1,2-dioleoyl-sn-glycero-3-phosphocholine; DANCR, Differentiation Antagonizing Non-Protein Coding RNA; DODAP-1,2-dioleoyl-3-dimethylammonium-propane; infMo, inflammatory monocytes; Lcn-2, Lipocalin-2; MIF, macrophage migration inhibitory factor; MSNP, mesoporous silica nanoparticle; mRNA, messenger RN; MTDH, metadherin; NP, nanoparticle; PAGA, Polyaminolated glycidyl methacrylate; PDPA, poly(2-(diisopropyl amino) ethyl methacrylate; PEG, Polyethyleneglycol; PEI, polyethyleneimine; Pgp, P-glycoprotein; PIGF, placental growth factor; PLK, Polo-like kinase; PLGA, poly (lactic-co-glycolic acid); PTPN, protein tyrosine phosphatase non-receptor; TAM, tumor-associated macrophage; T-Ly, T-cell; TME, tumor microenvironment; VEGF, vascular endothelial growth factor. Picture of breast cancer used from Servier Medical Art under CC-BY-3.0 license.

***Small interfering RNA (siRNA)*** is a species of the double-stranded non-coding RNA molecule. It is alternatively named short interfering RNA or silencing RNA because it operates within the RNA interference (RNAi) pathway. siRNAs, similar to miRNAs, interfere with the expression of target genes by binding to transcribed mRNA and inducing its degradation. siRNAs are produced within the nucleus from longer precursor dsRNAs (comprising 30 to over 100 nucleotides) and small hairpin RNAs. This is achieved via cleavage by endoribonuclease (RNA helicase) type III Dicer (as part of the RISC complex), which produces a short (20–24 bp in length) double-stranded interfering RNA (dsRNAs) with phosphorylated 5′ ends and hydroxylated 3′ ends and two overhanging nucleotides. The molecule is split later into single-stranded siRNAs (ss-siRNA). Like miRNAs, siRNA molecules bind to specific nucleoproteins (RNPs) from the Argonaute family (Ago), forming an RNA-induced silencing complex (RISC). The RISC-siRNA complex binds to complementary motifs of the target mRNA, and the mRNA strand is degraded by fragmentation and/or poly(A) tail shortening (Figure 6A) [241].

***Function.*** In comparison with miRNA, which demonstrates the lower complementarity and parallel targeting of multiple mRNAs (over 100 at the same time), the impact of siRNA is notably more selective, given its 100% complementarity with a specific target mRNA. Consequently, while the effects of siRNA action are largely characterized by a complete endonucleolytic cleavage of the targeted mRNA, this is less common for miRNAs, which typically exert their regulatory influence through mRNA degradation or physical binding to a target mRNA [241,242].

***siRNA in breast cancer.*** A majority of over 80% of potential therapeutic targets listed in worldwide cancer genome databases are currently inaccessible via conventional means of treatment such as monoclonal antibodies and small molecule inhibitors [243]. Therefore, RNA interference with exogenous synthetic siRNAs represents a significant potential for advancement in the field of cancer therapy. Since its discovery in 1998, siRNA has emerged as a highly valuable tool for the targeted knockdown of specific genes in cells. The introduction of siRNA into the body cells enables the silencing of any gene responsible for the various hallmarks of cancer, including angiogenesis, invasion, and metastasis. The first siRNA drugs were approved for clinical use by the US Food and Drug Administration and the European Medicines Agency between 2018 and 2022 [244]. For RNA interference to achieve its therapeutic potential, siRNA must be delivered to the site of action within the cells of target tissues. One significant challenge to the clinical implementation of siRNA-based therapeutics is the development of an effective delivery system, as siRNA oligopeptides are susceptible to rapid degradation by plasma and tissue endonucleases and exonucleases and exhibit a remarkably limited blood circulation time. Alternatively, the protection of siRNAs can be achieved by encapsulation within suitable nano-scale delivery vehicles (nanovectors), which permit diffusion between cells and afford protection against degradation (Figure 6B) [241,245,246]. Following successful cellular uptake (e.g., by receptor-driven endocytosis) the nanoparticles (NPs) comprising the siRNA decompose and undergo endosomal escape. The siRNA that is released into the cytosol interacts with the targeted mRNA and reversibly silences the gene expression via RNA interference (RNAi). Embedded therapeutical compounds can target required structures (Figure 6B).

It is commonly assumed that siRNAs predominantly downregulate and thereby silence the genes to which they are targeted. However, there are circumstances under which the application of synthetic siRNAs can yield opposite results, that is to say, they can lead to the upregulation of genes that have been targeted or unintended. It is hypothesized that this phenomenon is the consequence of an oversaturation of the RNAi machinery, which results in the depression of endogenous miRNA-regulated genes. Such facts must be subjected to careful consideration in all therapeutic implications of siRNA to circumvent any potential toxicity [241].

In recent years, a variety of nanoparticles and methods have been employed to harness the therapeutic potential of siRNA. Transfection applies to the majority of cell types and is characterized by high efficiency and reproducibility. siRNA that is designed to target a specific gene is typically delivered to the cell via cationic liposomes, lipid conjugation, or polymer nanoparticles, such as PEI or PEG. Viral delivery can be achieved through a variety of recombinant viral vectors based on retroviruses, adeno-associated viruses, adenoviruses, or lentiviruses. Viral vectors have been synthesized for the effective facilitation of siRNA that is not amenable to transfection into cells. Electroporation employs electrical pulses to facilitate the intracellular delivery of siRNA into cells. The number of carriers and strategies employed are thoroughly reviewed in previous works [247,248,249].

The utilization of siRNAs in therapeutic strategies can be delineated into three principal categories (Figure 6C). Firstly, siRNAs can be employed to emulate natural processes, specifically, the “silencing” of undesirable genes, which serves as a direct anti-tumor action. In terms of tumor progression and potential side effects, metastatic breast cancers and aggressive phenotypes, such as TNBCs, represent optimal targets (Figure 6C; see abbreviations). For instance, lipid-substituted polyethyleneimine (PEI) nanoparticles containing siPTPN22 were observed to effectively inhibit the PTPN22 gene, resulting in the highest percentage of suppressed cell migration in metastatic BC lines MDA-MB-231 and MCF7 [250]. Furthermore, PEG-b-PAGA-b-PDPA micelles (poly (ethylene glycol- block- poly(aminolated glycidyl methacrylate)-block- poly (2-(diisopropyl amino) ethyl methacrylate)) carrying siRNA against p65 (nuclear factor NF-κB P65 subunit)) demonstrated efficacy in inhibiting both primary breast tumors and lung metastasis when combined with cisplatin [251]. Furthermore, the combination of PEI-p-PDHA/PEG-PDHA micelles containing siTwist1 (a transcription factor) with paclitaxel demonstrated efficacy in suppressing the growth of 4T1 tumors and their metastasis [252]. TNBC is the most aggressive BC subtype. As it lacks hormone receptors (ER, PR) and HER2, the development of an appropriate targeted therapy is a significant challenge. However, the combined application of chemotherapy and antibody-siRNA nanoconjugates, in addition to the targeting and downregulation of one or more desired genes, has been demonstrated to reduce BC progression [246]. Recent studies employing siRNA-based therapy for TNBC appear to offer promising results. In a recent study, Ferreira and colleagues reported the use of an exosome-based system for siRNA loading (iExoMEK1) to downregulate the MAPK/ERK cascade (mitogen-activated protein kinase), which led to tumor regression and a reduction in angiogenesis in TNBC [253]. In a recent study, Nicolescu and colleagues employed dual-targeted ECO/siDANCR nanoparticles to target and silence an oncogenic lncRNA known as DANCR (differentiation antagonizing non-coding RNA) in Hs578T TNBC cells and MCF-7 cells [254]. Angiogenic-transcription-factor-SRY-related HMG-box 18 (SOX18) is significantly overexpressed in many cancers including human BC. The in vitro transfection of MCF-7 BC cell lines with siRNA against SOX18 inhibited cell proliferation and invasion and promoted apoptosis in breast cancer cells via the suppression of the Ras-dependent pathway [255]. Lipocalin 2 (Lcn2) overexpression in metastatic BC has a principal role in progression by inducing the epithelial-to-mesenchymal transition and enhancing tumor angiogenesis. PEGylated liposomes decorated with octreotide (OCT) peptide containing siRNA against Lcn2 for selective targeting BC cell line MCF-7 and TNBC cell lines could successfully reverse metastatic progression [256].

Another application of synthetic siRNAs is *to enhance the efficacy of conventional therapeutic agents*, including drugs and monoclonal antibodies. For example, MSNP-PEI-PEG nanoparticles with si-HER2 (against the mRNA of HER2) have been demonstrated to inhibit the growth of breast cancer HCC1954 lineages, which are otherwise unresponsive to anti-HER2 antibodies (trastuzumab) [257]. Yang et al. demonstrated that the use of siRNA against metadherin (MTDH) resulted in a notable enhancement in the efficacy of paclitaxel upon co-delivery by PEI-PLGA NP in an orthotopic tumor model (MCF-7) [258]. The co-delivery of siPgp (P-glycoprotein) and doxorubicin in MSNP-PEI-PEG -composed NPs has been demonstrated to enhance drug uptake by cancer cells [259]. The CXC Chemokine Receptor type 4 (CXCR4) has been demonstrated to play a pivotal role in the chemotactic invasion of cancer cells to secondary metastatic sites. CXCR4 is a druggable target that can be accessed through the use of antibodies or other molecular agents. Consequently, Zhou et al. have combined siRNA against vascular endothelial growth factor (VEGF) with CXCR4-binding cyclam polyplex to inhibit cancer cell (4T1 cell) invasion [260]. Moreover, the inhibition of CXCR4 expression via chitosan nanoparticle-delivered siRNA has been demonstrated to enhance the sensitivity of cells to cisplatin treatment in vitro [261].

Thirdly, the utilization of siRNA nanocomplexes has been evidenced to be an efficacious methodology for modulating the immunological surveillance of breast cancer by silencing specific genes. A variety of siRNA targets and NP vehicles were employed, with some examples illustrated in Figure 6. For example, Shen et al. [262] employed a siCCR2 (CC Motif Chemokine Receptor 2) embedded in PEG-PLA nanoparticles to silence the CCR2 gene, resulting in the generation of CCR2 null inflammatory monocytes and the subsequent inhibition of primary and metastatic BC. The knockout of CD73 via siCD73 in chitosan-lactate NPs has been demonstrated to enhance the efficacy of BC vaccines, thereby inhibiting tumor growth and lung metastases in 4T1 ectopic tumors [263]. It was demonstrated that the use of glucan NPs containing siRNA targeting MIF (macrophage migration inhibitory factor) inhibits 4T1 cancer cells [264].

The potential of siRNAs is being increasingly researched, with their potential in gene therapy and targeted treatment approaches constantly increasing [265].

### 3.6. Small Nuclear RNAs

***SnRNA (short nuclear RNA) or spliceosomal RNA*** is a diverse class of small single-stranded non-coding RNAs located within the subnuclear structures, specifically, Cajal bodies and splicing speckles. The average length of snRNA is 150 nt; snRNA molecules are always linked with specific proteins creating small nuclear ribonucleoproteins (snRNPs) or spliceosomes responsible for the precise removal of introns from pre-mRNA transcripts of the protein-coding genes [266,267,268]. Based on their conserved sequences and associated protein co-factors, snRNAs can be categorized into Sm class transcribed by RNA polymerase II (U1, U2, U4, U4atac, U5, U7, U11, and U12) or LSm class transcribed by RNA polymerase III (U6 and U6atac.) [269]. SnRNAs are formed from intronic parts of primary ss pre-mRNA transcripts (catalyzed by *RNA* polymerase II) that are not spliced and the 3′ end is not phosphorylated to prevent translation. They acquire their specific secondary structure and are embedded in complexes with specific snRNPs (small nuclear ribonucleoproteins) and begin their action in spliceosomes. SnRNAs are highly expressed in humans during proliferation (cell cycle) and cell development and are usually found in clusters in the genome [266].

***Function.*** It is estimated that over 90% of human protein-coding genes generate multiple mRNA isoforms, resulting in a protein isoform count that is at least five to ten times greater than the number of proteins encoded by the genome. This process is critically dependent on a well-functioning alternative splicing machinery, with small nuclear RNAs playing a central role. snRNPs are involved in processes regarding pre-messenger RNA processing and splicing, thereby creating a diverse pool of protein isoforms [270].

**Figure 7:**
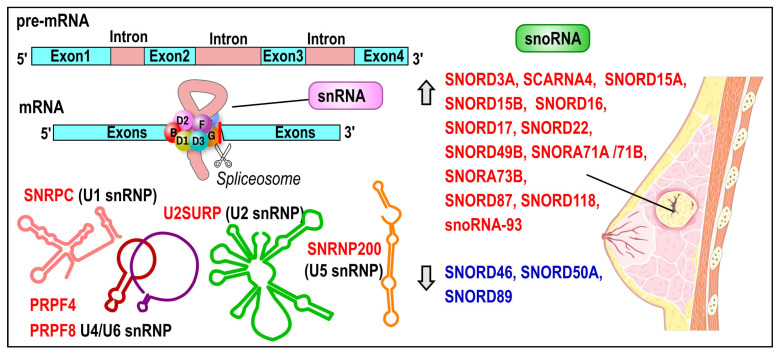
Small nuclear (snRNA) and small nucleolar (sncRNA) molecules in breast cancer (BC). snRNA is a class of small nuclear RNA molecules which complex with small nuclear ribonucleoproteins (snRNP) to form various types of spliceosomes. These are involved in the post-transcriptional slicing of introns from the pre-messenger RNA (*pre-mRNA*) to form a mature mRNA (*left panel*). Certain components of snRNP and snRNA, e.g., U1, U2, U4, U5, and U6 (illustrated in color), were found to be overexpressed (*red*) in BC. The *right panel* shows sncRNAs that are upregulated (*red*) or downregulated (*blue*) in different types of breast cancer. *Abb. PRPF4*, core component of U4/U6 snRNP; *PRPF8*, core component of U4/U6-U5 tri-snRNP spliceosome complex; *SNRPC*, small nuclear ribonucleoprotein polypeptide C; *SNRNP200*, U5 small nuclear ribonucleoprotein. See the text for further explanation.

***SnRNA in breast cancer.*** Koduru et al. 2017 identified 28 snRNAs and 123 snoRNAs that were dysregulated in triple-negative breast cancer (TNBC) tissues, indicating their involvement in cancerogenesis [271]. Small nuclear ribonucleoprotein polypeptide C (SNRPC), a subunit of U1 snRNP, has been connected to the acquisition of oncogenic character in TNBC cancer cells. The deletion of SNRPC significantly reduced their proliferation and invasiveness abilities both in vivo and in vitro, partially through the regulation of the TNFAIP2-Rac1-β-catenin signaling pathway [272] (Figure 7). *U2SURP* (U2 snRNP-associated SURP motif-containing protein) is upregulated in TNBC tissues and is associated with poor prognosis. The overexpression of U2SURP is MYC- and eIF3D-mediated and leads to increased oncogenic potential by promoting alternative splicing of SAT1 pre-mRNA [273]. SNRNP200 is a U5 small nuclear ribonucleoprotein 200 kDa helicase that promotes metabolic reprogramming and abnormal metabolism in TNBC cancer cells by enhancing glycolysis and glutathione metabolism. Splicing dysregulation leads to elevated glucose and nucleic acid metabolism in cancer cells [274]. The U4/U6-U5 tri-snRNP spliceosome complex and its core component PRPF8 are differently expressed between precancerous and breast cancer tissues. PRPF8 is overexpressed in breast cancer tissues and promotes cancer cell growth via the JAK-STAT signaling pathway and the inhibition of p21 [275]. Similarly, PRPF4, a core component of U4/U6 snRNP, is overexpressed in breast cancer cell lines and promotes cancer progression via p38 MAPK phosphorylation [276]. The overexpression of U6 snRNA in the serum of BC patients may be also a potential diagnostic biomarker [277].

### 3.7. Small Nucleolar RNAs

***Small nucleolar RNAs (snoRNAs)*** are one of the most abundant highly conserved classes of small non-coding RNA molecules located in the cell nucleolus. The average length of snoRNAs is 60–300 nucleotides [278]. In humans, most snoRNAs are splits of the intronic part of the primary mRNA transcript of protein-coding genes. Some snoRNAs are transcribed from non-coding intergenic dsDNA. Mature snoRNA molecules associate with core proteins to form an RNA/protein complex referred to as small nucleolar ribonucleoprotein particles (snoRNPs) [278,279], which are involved as a guide of the sequential chemical modification of various RNAs. Every snoRNA molecule contains an antisense element (a stretch of 10–20 nt) that is complementary to sequences in targeted RNA [279]. Two main classes of snoRNA are recognized: so-called *C/D box* (mostly associated with the methylation of target genes), and the *H/ACA box*, which is associated with the pseudouridylation of target molecules [280,281].

***Function.*** snoRNAs have several roles in cellular processes: (a) snoRNAs provide guidance in the synthesis and post-transcriptional chemical modification of other small non-coding RNA molecules like ribosomal RNAs, transfer RNAs, and small nuclear RNAs [279]. After transcription, pre-rRNA molecules undergo a series of processing steps to produce mature rRNA, such as methylation and pseudouridylation, which are directed by snoRNAs [282]. (b) snoRNAs can act as precursors for miRNA production. For example, the snoRNA ACA45 can be processed by the RNAse III enzyme Dicer into a mature miRNA with a length of 21 nt. (c) snoRNAs participate in the regulation of the alternative splicing of transgene transcripts (snoRNA HBII-52, also known as SNORD115).

***snoRNA in breast cancer.*** SnoRNAs are characterized by their stability in body fluids and their clinical relevance and represent promising tools as diagnostic and prognostic biomarkers. Alterations in snoRNA expression can affect numerous cellular processes, including cell proliferation, angiogenesis, fibrosis, and inflammation, making them a promising target for the diagnostics and treatment of various human pathologies. In recent years, data have been accumulated which show that the dysfunction of snoRNAs plays a pivotal role in tumorigenesis and related disorders [283].

Breast cancer is associated with a high overexpression of SNORD15A, SNORD15B, SNORD22, SNORD17, and SNORD87. Pro-malignant tumor behavior leading to metastatic breast cancer was demonstrated in SNORA71A/71B, SNORD3A, and SNORD118, and can imply the inhibition of the p53 tumor suppressor gene. Conversely, the expression of SNORD46 and SNORD89 is significantly decreased in breast cancer tissues. SNORD50A delayed the proliferation of breast cancer and improved prognosis [284,285] (Figure 7).

snoRNAs may be potential regulators of breast cancer cell growth via PARP-1’s catalytic activity, providing alternative therapeutic action without the DNA damage activation of PARP-1 [286]. snoRNA U50A may serve as a possible tumor suppressor gene as it is correlated with better overall survival in breast cancer patients. Both in vivo and in vitro, U50A prolongs mitosis and reduces its colony-forming ability [287]. Recently, the same group of authors provided data which show that snoRNA U50A can decrease the sensitivity of breast cancer cells to everolimus treatment by downregulating mTOR gene expression, possibly via suppression of the c-Myc [288]. Four snoRNAs, SNORD16, SNORA73B, SCARNA4, and SNORD49B, have been coined as possible early-stage diagnostic markers of breast cancer as they were more abundant in both the cancer tissue and serum of breast cancer patients [289]. snoRNAs contribute to the oncogenic character of breast cancer cells through their procession into small nucleolar RNA-derived RNAs, which are short microRNA-like fragments. The overexpression of sdRNA-93 has been shown to cause increased invasiveness; the most robust overexpression was proven in luminal B HER2+ tumors [290]. SNORA71A and SNORA71B are both overexpressed in metastatic breast cancer tissue. SNORA71A promotes cell proliferation, epithelial–mesenchymal transition, and invasiveness by binding to G3BP1 and stabilizing ROCK2 [291]. SNORA71B promotes brain metastasis in breast cancer and blood–brain barrier cancer cell migration partly via inducing epithelial–mesenchymal transition [292].

Our comprehension of the function of small nucleolar RNAs (snoRNAs) and small nuclear RNAs (snRNAs) in the modulation of gene expression and their prospective utility in the management of breast cancer (BC) is continuously expanding [293,294].

## 4. Clinical Diagnostic and Therapeutic Use of Non-Coding RNAs in Breast Cancer

Non-coding RNAs are currently emerging as modern biomarkers for many diseases, and our review focuses on their role in breast cancer. Breast cancer is routinely diagnosed using non-invasive imaging methods such as ultrasound, mammogram, and magnetic resonance imaging. Nevertheless, the biopsy, as an invasive method, remains the fundamental approach for the current diagnosing, classifying, and staging of a tumor [295]. If sufficiently sensitive, specific, accurate, and reproducible ncRNA-based diagnostics may provide many advantages. NcRNAs can be obtained easily and more or less non-invasively from blood and other body fluids such as saliva, tears, and urine, and they can be obtained in early stages of cancer or as a preventive measure [296].

According to recent studies discussed in previous chapters, non-coding RNAs’ genes are deregulated in human cancers (e.g., via deletion, amplification, and abnormal epigenetic or transcriptional regulation) and they further deregulate a wide range of dependent downstream genes and related signaling pathways [297]. Precise knowledge on how individual ncRNAs regulate gene expression on post-transcriptional and post-translational levels (interaction network among ncRNAs, ncRNA and mRNA, and ncRNA and proteins) is inevitable for the future development of effective ncRNA therapeutics. As such, ncRNAs represent both therapeutical targets as well as potential therapeutic tools. From the current perspective, these could include ***(a) replacement (facilitating) therapies*** injecting into the body synthetic (s) ncRNA such as s-miRNAs, short hairpin RNAs (shRNAs), s-siRNAs, s-piRNAs, s-circRNAs, s-lncRNA, s-snoRNAs, and also s-tRNAs or miRNA mimics, and ***(b) inhibitory (blocking) therapies*** such as antisense oligonucleotides (ASOs) to block lncRNA or miRNAs (ASO-anti-microRNAs; antimiRs), miRNA sponges, miRNA inhibitors, and CRISPR/Cas9-based gene machinery (clustered regularly interspaced short palindromic repeats/CRISPR-associated protein 9) for editing genes including miRNA, lncRNA, and snoRNA [297,298,299].

In order to protect fragile and easily degradable ncRNA biomolecules during local or systemic applications, to enhance tissue penetration, depotting, and other pharmacokinetic properties, numerous innovative delivery strategies have been in development for an extended period. Such carriers or vectors may be composed of, for example, the following: (a) liposomes (comprising hydrophilic cores encased in lipid bilayers of a hydrophobic nature), (b) endosomes (natural vesicles measuring 40–150 nm in diameter serving as the transportation of miRNAs or siRNAs), (c) peptide transmembrane carriers (e.g., pHLIP for anti-miRs), (d) cationic polymers and similar artificial multilayered particles, (e) solid lipid nanoparticles, and, finally, (f) viral vectors, of which examples include adeno-associated viruses (AAV), lentiviruses, and retroviruses [297,298]. The use of most of these methods is still in the preclinical stage.

### 4.1. LncRNA in the Diagnostics and Therapy of Breast Cancer

LncRNA represent the largest and most diverse class of non-coding transcripts with up to 60,000 lncRNA genes present in the human genome. They are capable of regulating gene expression at the genomic, transcriptomic, and proteomic level, and, due to their interaction domains for DNA, mRNA, miRNA, and proteins, they can act as antisense transcripts and regulate the expression of their opposite sense transcripts [300]. The result is transcriptional interference, which is influenced by competition for transcription factors and RNA polymerase II or by facilitating DNA methylation through histone modification. They have been implicated in a variety of cellular functions including epigenetic gene regulation, acting as decoys or “sponges” for miRNAs, acting as molecular scaffolds, splicing, mRNA stability, and translation [301]. Specific lncRNA signatures detected so far for various subtypes of breast cancer in vivo in animal models or in humans significantly improved the predictably, prognosis, and stratification of BC patients for chemotherapy and radiotherapy. The diagnostic value of lncRNAs is further verified by running clinical trials (Table 3). Other comprehensive reviews further explore the potential of lnc-RNA as a diagnostic biomarker and its applications in BC treatment [75].

Long non-coding RNAs represent a promising target for cancer therapy. Recently, Li et al. reviewed current candidate lncRNA for targeted therapy including MALAT1 (with both an oncogenic and tumor-suppressive role), TYMOS, GATA3-AS1, TINCR, SNHG16, and lncRNA Xist (all promote immune escape or immunosuppression) [302].

The inactivation of carcinogenic lncRNA may be achieved by targeting it with ASO, which may trigger RNase H-mediated RNA degradation [300]. Antisense RNA (aRNA) are small diffusible ncRNAs, oligonucleotides of 19–23 nt length, produced normally in the cells during DNA transcription which bind to complementary mRNA motifs. It is estimated that 30% of human protein-coding genes have a corresponding antisense RNA. Currently, ASOs or their bicyclic RNA analogues (locked nucleic acids, LNAs) are the most commonly used method for targeting specific miRNAs or, eventually, lncRNAs. Culbertson et al. identified an example of an antisense RNA that, according to their experiment in a mouse model, contributes to the metastasis of breast cancer cells and resistance to oxidative stress. This RNA is complementary to the 3′-UTR of NADPH quinone dehydrogenase 1, and was, therefore, coined as NQO1-AS. NQO1-AS stabilizes NQO1 and increases the amount of its gene product [303]. Another specific sense/antisense pair is the oncogene MACC1 and the lncRNA MACC AS-1. The binding of MACC AS-1 to the MACC1 promotor triggers a cascade of reactions (binding of DEAD-Box helicase 5 (DDX5) to the MACC1 promoter, activation of the transcription factor SP-1) which lead to the promotion of MACC1 gene expression. The increased expression of MACC1 leads to the increased proliferation of breast tumor cells [304]. The modulation of the amount of sense transcripts of oncogenic genes could be another therapeutic tool at the molecular level. Another technology that has been proposed as a means of targeting lncRNAs as a cancer therapeutic strategy is CRISPR-Cas9 editing. However, the delivery of CRISPR-Cas9 and the targeting of cells are more problematic, requiring the use of viral vectors [297,299]. Currently, there are no such registered clinical trials for BC. The current stage of knowledge and further possibilities of lncRNA in the diagnosis and treatment of cancer were recently reviewed [305,306].

### 4.2. miRNA in the Diagnostics and Therapy of Breast Cancer

MicroRNAs were among the first ncRNAs investigated as diagnostic and prognostic biomarkers, and their possible role as therapeutic targets is thoroughly investigated as well [298]. Dziechciowska and collaborators recently proposed the most suitable candidates for critical diagnostic and therapeutic miRNA targets in BC. Among the important upregulated miRNAs analyzed (qRT-PCR) from serum or tissues, they found miR-21 (serum), miR-9 (cell culture), and miR-155 (serum). The list of important downregulated miRNA candidates includes let-7c (serum), miR-101 (tissue), miR-126 (tissue), miR-185-3p (tissue), miR-141 (tissue), miR-199a (tissue), and miR-335 (serum) [307]. Several clinical trials evaluating the diagnostic significance of miRNAs were recently finished or are currently underway (Table 4). The principal objective of these trials is to ascertain the validity of miRNA signatures associated with specific breast cancer subtypes with a view to improving treatment response and prognosis.

With regard to the therapy, two principal strategies may be employed to manipulate gene expression and modulate the quantity of mRNA through miRNAs: the reintroduction of miRNA or downregulation (inhibition) of miRNA in cancer cells. The ectopic overactivity of specific miRNAs may be achieved through the utilization of vectors that overexpress the target miRNA or the employment of miRNA mimics. Irrespective of the method employed, the reintroduced miRNA should be capable of achieving the same biological functions as the endogenous miRNA, including the silencing of the target mRNA. It is expected that synthetic miRNAs will be able to be loaded into the RISC complex and to recognize and then interfere with the mRNA targets [298]. ***miRNA mimics*** are mostly synthetic double-stranded oligonucleotides composed by both passenger and guide strand for a better silencing effect due to the more efficient loading of the RNA molecule in the RISC complex. For instance, miR- 203 mimic, miR-34a mimic (drug MRX34), miR-143, and miR-145 mimics show tumor suppressor activity across different cancers [297,298,302].

***miRNA inhibitors*** are designed to inactivate the upregulated expression of onco-miRs in cancer cells (e.g., inhibitor of miR-21 in many cancers, miR-10b in BC, or miR-155 (Cobomarsen in leukemias)) [302,306]. Another common method for inhibiting specific miRNAs is to design antisense oligonucleotides (ASOs; ASO antionco-miRs) complementary to the target-specific miRNAs that inhibit their function (e.g., against miRNA-214, miRNA-31 in gastrointestinal tumors, or miRNA-21 in BC) [297,298]. The ***miRNA mask*** is another strategy achieved by competing with the binding of endogenous miRNA on the 3′UTR of the target mRNA and the consequent degradation by RISC complex, leading to a relief of translational repression [297,302]. A clinical study by Hedayat et al. from 2024 confirmed the role of miR-652-3p in resistance to the multikinase inhibitor regorafenib, which slows down the growth, proliferation, and spread of BC cells. miRNA inhibitors inactivate miR-652-3p, increase efficacy of regorafenib and improve treatment [302,308]. Another application of miRNA inhibitors is the targeting of miRNAs that are involved in the formation and maintenance of cancer stem cells (CSCs). CSCs are tumor cells with normal stem cell properties which contribute to the growth of the tumor mass (through the process of stem cell self-renewal and differentiation into multiple cell types) [309]. miRNAs contribute to the establishment and maintenance of the cancer stem cell phenotype by the abnormal activation or inhibition of specific signaling pathways: Notch (miR-9 targeting Notch1), PI3K/Akt (miR-10b, miR-21, miR-181c targeting PTEN), and NF-κB (miR-181 targeting PHLDA1) [310].

Despite the large number of miRNAs analyzed as possible therapeutics, only a few were recently in clinical trials as anticancer therapies, e.g., TargomiRs targeting miR-16 for non-small-cell lung cancer or MRX34 targeting miR-34a for the treatment of melanoma, primary liver cancer, and other tumors. MRX34, a synthetic mimic of the highly studied tumor suppressor miR-34a, suppresses metastasis and stemness in various cancers including BC. A clinical trial with MRX34 was halted due to immune-related adverse effects [297]. Another example was a clinical study (NCT04675996) investigating the effect of the drug INT-1B3. This is a lipid nanoparticle (LNP)-formulated miR-193a-3p mimic (1B3), the effect of which was also studied in TNBC-derived cell lines. The result so far is the observation of the upregulation of the PTEN signaling pathway and the downregulation of oncogenic signaling pathways, which led to the inhibition of tumor cell growth and proliferation. The study was terminated in Phase I [311]. No such clinical trials are currently registered for breast cancer [298].

### 4.3. siRNA in the Therapy of Breast Cancer

siRNA-based therapies have been developed for the past 20 years. To date, the FDA has approved 6 siRNA agents (patisiran, givosiran, lumasiran, inclisiran, nedosiran, and vutisiran), all for non-oncology treatments, and more than 20 siRNA therapeutics have entered clinical trials. As a therapy, siRNA is able to be delivered locally, through the eye or nose, to treat various diseases. Local delivery benefits from a simpler design and easier drug delivery and the much higher bioavailability of the drug. The systemic delivery of synthetic siRNA (s-siRNA), typically via intravenous injection, is used to target deep tissues including cancers. Such an application, however, has proven challenging due to weak cellular uptake, often requiring very high doses to saturate the tissues, and the very short blood half-life time (a few days or a few weeks at most) [265].

Synthetic siRNAs are the most suitable for use as drugs due to their unique advantages over other anti-cancer agents: (a) siRNAs can recognize the target gene with high specificity due to the base pairing recognition. (b) They exert their post-transcriptional gene silencing in the cytoplasm, thus minimizing the risk of host gene mutations. (c) siRNAs show remarkable efficiency as, even with few fragments, siRNAs can induce significant gene silencing effects in cells. Several clinical trials using siRNA have been completed or are currently underway in numerous tumor indications (e.g., liver cancer 2, colorectal cancer 3, pancreatic cancer 7, lymphoma 4, solid tumors, renal cancer 2, prostatic cancer 2, melanoma 2, gastric cancer 1, lung carcinoma 1) [298]. A limited number of clinical trials have employed s-siRNA in the context of breast cancer, albeit not as a principal objective (see Table 5).

CALAA-01 was one of first siRNA nanotherapeutics introduced into clinical tests (2008–2013) as a targeted therapy for solid tumors including breast and prostatic cancer, respectively. Nanoparticles consisting of cyclodextrin-containing polymer (CAL101), stabilizing agent (AD-PEG), targeting agent (AD-PEG-Tf), and human transferrin protein contained synthetic siRNA against the mRNA of the M2 subunit of ribonucleotide reductase (R2). Tests were terminated due to certain side effects [265,312]. Clinical trials were further conducted with the intravenous application of nanodrug TKM-080301 in breast and ovarian cancers with hepatic metastasis (NCT01437007) (Table 3). TKM-080301 contained siRNA against Polo-like kinase 1 (PLK1), a serine–threonine protein kinase that is involved in cell division and DNA damage and is overexpressed in many solid tumors including TNBC [313,314]. Anti-PLK1 siRNA was embedded in 120 nm sized SNALPs (Stable Nucleic Acid Lipid Particles) composed of mixture of cationic and fusogenic lipid layers and diffusible polyethylene glycol. Clinical trials were conducted preferentially in solid tumors such as hepatocellular carcinoma and pancreatic and colorectal cancer, but demonstrated average therapeutic response [265,315]. Nevertheless, mitotic kinases, such as PLK1, due to their role in intracellular signaling, remain the prospective target of future anticancer therapy, particularly in metastatic forms of BC [316]. PLK1 inhibitors such as BI2536 reached clinical trials but were terminated because of a poor therapeutic index [314]. Other delivery systems for anti-PLK1 siRNA to be utilized specifically in BC cell lineages have been recently tested using a 50 nm mesoporous silica nanoparticle (MSNP) core coated with bioreducible cross-linked PEI and PEG polymers [313]. One of currently running clinical trials is testing the safety and tolerability (toxicity profile) of siRNA against the mRNA of EphA2 (Tyr-kinase receptor 2 for ephrin A), which is encapsulated in neutral liposome DOPC (1,2-dioleoyl-sn- glycero-3- phosphatidylcholine) EphA2- siRNA is applied intravenously in patients with advanced/recurrent malignancies (NCT01591356) (Table 5). EphA2 regulates tumor-related neoangiogenesis, which is necessary for tumor maintenances through the PI3K signaling pathway EphA2 protein and mRNA, which are both highly expressed across multiple molecular subtypes of breast cancer (positive rate ~75% and ~84%, respectively) [317]. The results are of interest as a high level of EphA correlates with poor patient prognosis in metastasing BC [318].

### 4.4. circRNA in Diagnostics of Breast Cancer

Due to their structural stability, circRNAs can be easily measured in biological fluids such as blood, urine, and saliva, can be isolated from exosomes, and can be extracted directly from tumor tissue. Therefore, research into circRNAs has attracted considerable attention in recent years. As previously outlined, breast cancer and its phenotypes are associated with the overexpression or underexpression of numerous distinct circRNAs (dozens or hundreds). circRNAs carry a substantial number of complementary sequences for binding one or more miRNAs, thereby functioning as effective competitive endogenous RNAs (ceRNAs). circRNAs act as molecular sponges that can bind miRNAs, which are unable to bind on their target mRNAs or lncRNAs. Therefore, circRNAs have become the cornerstone of the now well-established circRNA–miRNA–mRNA regulatory axes in RNA regulatory networks. They are proving to be reliable diagnostic and prognostic markers that allow for the prediction of the distinctive characteristics of the cells that are predominant in a particular tumor. In a recent updated review, Bao et al. highlighted the role of approximately 30–40 circRNA–miRNA–mRNA regulatory axes in human breast cancer. These circRNAs exert stimulatory-oncogenic (28 detected) or inhibitory-suppressive (7 cases) effects on various tumor characteristics: proliferation, invasiveness, migration, metastasis, survival, immune escape, and drug resistance [319]. Undoubtedly, one of the highest overexpressions of circRNA in malignant tumors was shown for circ-ABCB10, which targets miR-1271. In BC, its level is 5–10 times higher than in normal cells. The knockdown of circ-ABCB10 can lead to cell cycle arrest in the G0/G1 phase and the inhibition of BC proliferation in vitro [319]. In contrast, circVRK1, which targets miR-17 and the tumor suppressor PTEN, is highly underexpressed in BC and other malignancies [218]. Xu et al. recently selected 32 upregulated and 4 downregulated circRNAs and their sponged miRNAs and mRNA targets in patients with aggressive TNBC. Oncogenic circRNAs such as circCAPG, circDNAJC11, circSEPT9, circTBC1D14, circKIF4A, circRAD18, circEPSTI, or circFRA1 and tumor suppressor cicRNAs such as circAHNAK1, circTADA2A-E6, circCREIT, circ_0000977, circ_0044234, and circUSP42 show a very significant correlation with clinicopathological features and the TNM stage of TNBC [320]. circRNAs in exosomes (exo-circRNA) are highly expressed in BC and could be easily analyzed for diagnostic purposes from body fluid or tissue samples. Hussen et al. recently reviewed around 50 overexpressed exo-circRNA and 20 underexpressed exo-circRNAs isolated from breast cancer cell lines and surrounding tissues for future diagnostic purposes [321].

Of clinical interest are circRNAs that may signal an increased resistance of BC to chemotherapeutic agents, e.g., circEGFR (via miR-1299) to trastuzumab and pertuzumab, circUBE2D2 (via miR-512-3p) and hsa_circ_0092276 to doxorubicin, circGFRA1 (via miR-3615p) and cisABCB10 (via to let-7a-5p) to paclitaxel, and circUBE2D2 (via miR-200a) to tamoxifen [322]. In contrast, the tumor suppressor circDUSP1 (via miR-761) increases sensitivity to paclitaxel, circUBAP2 (via miR-300) increases sensitivity to cisplatin, and circBMPR2 (via miR-553) to tamoxifen [323].

### 4.5. Analysis of ncRNA and Other Methodologies in the Molecular Diagnostics of Breast Cancer

There are various options for analyzing non-coding RNA, but the most widely used methodologies are microarray and next generation sequencing (NGS). RNA molecules are isolated from a sample and further analyzed based on specific needs.

***Microarray*** is a convenient and relatively inexpensive technique for determining the amount of expression of a gene of interest. The amount of transcript is determined by the hybridization of fluorescently labelled transcripts to probes. Biological samples are arranged two-dimensionally and are located in individual wells in a solid phase, which are organized into rows and columns. Microarray techniques are suitable for comparing two groups of samples in order to determine whether given mutations or changes in RNA expression are associated with a particular phenotype. The advantage of microarray is the possibility of monitoring a large number of molecules originating from a single sample and this allows for a simple comparison of healthy individuals and patients within a single experiment [324]. In general, the disadvantage of analyzing ncRNAs is their smaller amount and size compared to mRNA (the exception is long non-coding RNAs); therefore, a more sensitive and selective approach to their processing and analysis is needed [325]. The disadvantage of microarrays may be a possible inefficiency due to the lower amount of detected transcripts hybridized with the corresponding probes, and the fact that probes must be designed only based on already-known sequences [326].

***Commercial tests*** can monitor the expression of selected genes and are designed to classify tumors, personalize treatment, and predict disease relapse. The advantage of these tests is the speed of the delivery of results, but a major disadvantage is the fact that they are only intended for women with early-stage cancer [327]. Tests such as MammaPrint and BluePrint monitor the expression of selected genes using a microarray platform. The MammaPrint test includes monitoring the expression of the 70 genes most responsible for the recurrence of the cancer within ten years after the successful completion of treatment. It allows for determining the body’s response to chemotherapy treatment, The BluePrint test monitors the expression of 80 genes and is aimed at determining the individual tumor profile and more precisely determining the category to which it will be classified [8,328].

***Next generation sequencing*** is a technology for massive parallel sequencing and DNA sequencing. NGS allows DNA to be sequenced more cheaply and quickly compared to older sequencing methods. A major advantage of NGS is the reduction in the amount of template and reaction components required. NGS allows for the simultaneous sequencing of a large number of different DNA sequences [329]. It allows for the identification of prognostic genes that help predict tumor behavior before any treatment is initiated and the predictive genes that predict the possible behavior of the tumor under specific types of treatment. NGS is particularly suitable for patients with metastatic breast cancer. NGS requires more sophisticated bioinformatics systems, computing power, and the ability to store large amounts of data, which may be impossible for many institutions [330].

## 5. Conclusions

Breast cancer remains one of the most prevalent causes of cancer-related deaths among women worldwide. Recent advances in breast cancer research and molecular biology have highlighted the crucial role of non-coding RNAs in tumorigenesis and, more specifically, in the pathogenesis, diagnosis, and therapy of breast cancer.

The significance of non-coding RNAs in cancer is a subject of extensive research, with a particular focus on their involvement in cellular processes at both the pre- and post-transcriptional level. Non-coding RNAs have been shown to act as vital regulators of gene expression and post-transcriptional modification, which, in turn, leads to the creation of a variety of protein isoforms. It has been demonstrated that non-coding RNAs regulate several pivotal biological processes that are involved in the development of tumors, including cell proliferation, apoptosis, metastasis, and drug resistance.

Due to their stability in bodily fluids, non-coding RNAs extracted from blood or other biological fluids have the potential to be utilized as biomarkers, facilitating the more precise and less invasive diagnostics and the more effective therapy of breast cancer. The further accumulation of knowledge on non-coding RNAs can support and promote the development of novel therapeutic strategies aimed at modulating their activity. Novel therapy approaches, such as the inhibition of dysregulated miRNAs or the targeting of lncRNAs by antisense oligonucleotides, may complement existing treatments, thereby improving patient outcomes and overcoming limitations such as resistance to conventional therapy.

## Figures and Tables

**Table 2:** The enRNA network components (circRNA–miRNA–mRNA) in breast cancer.

circRNA	miRNA	Protein Gene (mRNA)	Sources
**ER+/PR+ breast cancers**			
circPGR	miR-301a-5p	CDK1, CDK6, CHECK2	[207]
circ_0025202	miR-182-5p	FOXO3a	[198]
**HR − HER2+**			
circ-MMP11	miR-153-3P	ANLN	[199]
circ-MMP11	miR-1204	MMP11	[199]
circCDYL	miR-92b-3p	circCDYL	[200]
circNR3C2	miR-513a-3p	HRD1/Vimentin	[215]
circ_0009910	miR-145-5p	MUC1	[216]
**TNBC**			
circCDYL	miR-1275-ULK1	ATG7	[201]
circSEPT9	miR-637	LIF/Stat3	[204]
circGFRA1	miR-34a	GFRA1	[206]
circNR3C2	miR-513a3p	HRD1/Vimentin	[215]
circHIPK3	miR-124-3p	MTDH	[193]
circUBE2D2/	miR-512-3p	CDCA3	[211]
circFBXW7	miR-197-3p	FBXW7	[213]
circ_0000199	miR-206/613	PI3K/Akt/mTOR	[210]
circKIF4A	miR-375	KIF4A	[207]

**Table 3:** Registered clinical tests using lncRNA in breast cancer (data according to ClinicalTrials.gov).

ClinicalTrials.gov Identifier/Status	Title/Objectives (Notes)
NCT06307249 Recruiting	**Precision Therapy for Solid Tumors: Synergistic CDK4/6 Inhibition and Anti-VEGF Targeting LncRNA (PTST_PALBEVA)** Use of LncRNAs as biomarkers in solid tumors (including BC) in patients receiving Palbociclib (a cyclin-dependent kinase 4/6 (CDK4/6) inhibitor) or Bevacizumab (a vascular endothelial growth factor (VEGF) inhibitor).
NCT02641847 Active	**TA(E)C-GP Versus A(E)C-T for High-Risk TNBC Patients and the Validation of the mRNA-lncRNA Signature** mRNA-lncRNA integrated signatures to evaluate efficacy and safety of mono- or combined therapy (docetaxel, doxorubicin (epirubicin), cyclophosphamide, gemcitabine, cisplatin in TNBC).
NCT06357689 Completed	**Association of SNPs in Long Intergenic Non-Coding RNA 00511 (LINC00511) with Breast Cancer among the Egyptian Population** Role of LINC00511 SNPs (rs11657109 or rs17780195 or rs9906859, rs4432291 and rs1558535) in breast cancer susceptibility in the Egyptian population.
NCT06427720 Completed	**LINC00511/miR-185-3p Axis and miR-301a-3p Markers for Breast Cancer Diagnosis** Role of oncogenic miRNA-301, tumor suppressor miRNA mi-185-3p, and intergenic lncRNA (LINC00511) as diagnostic markers in metastatic breast cancer.

**Table 4:** Registered clinical trials using miRNAs in breast cancer (data according to ClinicalTrials.gov).

ClinicalTrials.gov Identifier/Status	Title/Objectives (Notes)
NCT06529614 Recruiting	**In Situ Detection for MicroRNA** New in situ detection method for MicroRNA in the serum of patients with breast cancer.
NCT06439940 Recruiting	**Oncoliq: Early Breast Cancer Detection Based on Liquid Biopsies and microRNAs** Novel early breast cancer detection test based on liquid biopsies and microRNAs in women volunteers without previous cancer diagnosis who go to the annual medical control.
NCT04906330 Recruiting	**Oncoliq: Test for Early Breast Cancer Detection** Novel early breast cancer detection test based on liquid biopsies and microRNAs.
NCT02253251 Recruiting	**Clinical Validation of the Role of microRNA Binding Site Mutations in Cancer Risk, Prevention, and Treatment** Using an miRNA panel in the characterization of sKRAS-variant BRCA-negative BC patients.
NCT05633342 Recruiting	**Project CADENCE (Cancer Detected Early Can be Cured)** Difference in miRNA expression levels between non-cancer and cancer groups combining multiomic cancer biomarkers.
NCT02656589 Unknown status	**microRNA of Human Epidermal Growth Factor Receptor 2 (HER2)-Positive Patient Treated With Herceptin** miRNA profiles in HER2+ stage IV BC patients with no history of chemotherapy, hormone therapy, radiotherapy, or surgery treated with Herceptin, Capecitabine, or trastuzumab.
NCT04771871 Unknown status	**MicroRNA Profiles in Triple-Negative Breast Cancer (TARMAC)** Blood levels of microRNAs and circulating tumor DNA in response to standard chemotherapy (Epirubicin, Cyclophosphamide, Paclitaxel, Carboplatin) in TNBC patients.
NCT04778202 Unknown status	**Diagnostic and Prognostic Value of MicroRNA in Breast Cancer Patients** Blood levels of miRNA 125a-5p and miRNA143-3p levels in diagnosis of breast cancer; correlations with histopathological data and MRI radiological findings.
NCT03779022 Unknown status	**miRNA and Relevant Biomarkers of BC Patients Undergoing Neoadjuvant Treatment** Selection of miRNA panel suitable for treatment of early breast cancer patients (Stage II-III dis.).
NCT04720508 Unknown status	**Aberrant Expression of Micro RNA for Diagnosis of Breast Cancer** Correlations of serum levels of miRNA-373, miRNA-425-5p with clinicopathological data (staging, grading, and tumor receptors) in patients with breast cancer.
NCT05151224 Unknown status	**Circulating microRNA 21 Expression Level Before and After Neoadjuvant Systemic Therapy in Breast Carcinoma** miRNA-21, a key oncomir in breast cancer before and after neoadjuvant therapy.
NCT02950207 Unknown status	**Prospective Observational Study of Antitumor Activity Correlation Between Hormonal Therapy and Expression miRNA100 (BC-P1-2013)** ID miRNA 100 as a predictor of hormone responsiveness in hormone-positive BC subtypes.
NCT04516330 Completed	**Can MicroRNAs Predict Multicentricity in Breast Cancer?** Search for a specific microRNA that helps identify multicentric breast cancer as compared multicentricity; it will help us to make appropriate decisions on the treatment of patients with breast cancer
NCT01231386 Completed	**MIRNA Profiling of Breast Cancer in Patients Undergoing Neoadjuvant or Adjuvant Treatment for Locally Advanced and Inflammatory Breast Cancer Conditions** miRNA profiles indicating therapeutic responses and serving as prognostic markers.
NCT03255486 Completed	**Identification and Evaluation of Biomarkers of Resistance to Neoadjuvant Chemotherapy** Profiles of biomarkers of resistance to in locally advanced breast cancers which are sensitive or resistant to neoadjuvant chemotherapy (locally advanced stage).
NCT01722851 Completed	**Circulating miRNAs** To identify a panel of miRNA markers in blood in new or recurrent BC patients to choose the best neoadjuvant and adjuvant chemotherapy, stratification, and prognosis.
NCT01612871 Completed	**Circulating miRNAs as Biomarkers of Hormone Sensitivity in Breast Cancer** Panel of 15 tissue microRNAs involved in hormonal therapy resistance/sensitivity was tested in metastatic ER+/PR+ BC or locally advanced tumor.
NCT02065908 Completed	**Circulating MicroRNA as a Biomarker of Cardiotoxicity in Breast Cancer** Blood miRNA profile in stage I-III TNM (Tumor–Node–Metastases) BC patients before and after anthracycline chemotherapy to indicate possible cardiotoxicity.
NCT06555354 Completed	**MicroRNAs and Prognosis in Breast Cancer (BREMIR)** Use of miRNAs (tissue, plasma) using GeneChip^®^ miRNA 4.0 array as predictors of developing metastases in BC patient receiving chemotherapy.
NCT01722851 Completed	**Circulating miRNAs** Panel of circulating miRNA markers to identify BC patients who are most likely to respond well to neoadjuvant and adjuvant chemotherapy.
NCT01598285 Terminated	**A Combined GWAS and miRNA for the Identification of Bevacizumab Response Predictors in Metastatic Breast Cancer** Searching for specific genetic variants (SNPs) and miRNA signatures associated with bevacizumab response in metastatic breast cancer patients.
NCT02127073 Terminated	**Pilot Study of Oxytocin and microRNA Identification in NAF, Serum, and Tissue in Women With Breast Cancer** miR fingerprint in NAF (nipple aspirate fluid), serum, and tissue in patients with ductal carcinoma in situ (DCIS) or invasive BC biomarkers.
NCT04675996 Terminated	**First in-Human Study of INT-1B3 in Patients With Advanced Solid Tumors** Efficiency of nanoparticle-formulated microRNA (miR-193a-3p) mimic destined for therapeutic intervention in oncology.

**Table 5:** Registered clinical trials using siRNA and circRNA in breast cancer (data according to ClinicalTrials.gov).

ClinicalTrials.gov Identifier/Status	Title/Objectives (Notes)
**siRNA**	
NCT01591356 Active	**EphA2 EPHARNA Advanced Malignant Solid Neoplasm** Safety, tolerability (toxicity profile), pharmacokinetic profile, and efficacy of intravenous siRNA-EphA2-DOPC.
NCT01437007 Completed	**TKM 080301 for Primary or Secondary Liver Cancer** Breast cancer with hepatic metastase.
NCT00689065 Terminated	**Safety Study of CALAA-01 to Treat Solid Tumor Cancers** Safety, toxicity, pharmacokinetics, and maximum tolerated dose (MTD) of intravenous CALAA-01 (siRNA) to patients with relapsed or refractory cancer.
**circRNA**	
NCT05771337 Not yet recruiting	**Circular RNA and Chemerin in Breast Cancer Patient** Diagnostic and prognostic values of chemerin, hsa_circ_0001785 (Circ-ELP3), and hsa_circ_100219 (Circ-FAF1) in correlation with CEA + CA15-3 in the serum of BC patients.
NCT06530082 Not yet recruiting	**A Single-Arm Clinical Study of Dendritic Cell Vaccine Loaded with Circular RNA Encoding Cryptic Peptide for Patients with HER2-negative Advanced Breast Cancer** Safety and tolerability of CircFAM53B-219aa DC vaccine monotherapy and its combination with camrelizumab in the treatment of HER2-negative advanced breast cancer.

## Data Availability

No new data were created or analyzed in this study.
